# Why the Early Paleozoic was intrinsically prone to marine extinction

**DOI:** 10.1126/sciadv.adg7679

**Published:** 2023-08-30

**Authors:** Alexandre Pohl, Richard G. Stockey, Xu Dai, Ryan Yohler, Guillaume Le Hir, Dominik Hülse, Arnaud Brayard, Seth Finnegan, Andy Ridgwell

**Affiliations:** ^1^ Biogéosciences, UMR 6282 CNRS, Université de Bourgogne, 6 Boulevard Gabriel, 21000 Dijon, France.; ^2^ School of Ocean and Earth Science, National Oceanography Centre Southampton, University of Southampton, Southampton, UK.; ^3^ Department of Integrative Biology, University of California, Berkeley, Berkeley, CA, USA.; ^4^ Université de Paris, Institut de Physique du Globe de Paris, CNRS, 1 rue Jussieu, 75005 Paris, France.; ^5^ Max-Planck-Institute for Meteorology, Hamburg, Germany.; ^6^ Department of Earth and Planetary Sciences, University of California, Riverside, CA, USA.

## Abstract

The geological record of marine animal biodiversity reflects the interplay between changing rates of speciation versus extinction. Compared to mass extinctions, background extinctions have received little attention. To disentangle the different contributions of global climate state, continental configuration, and atmospheric oxygen concentration (*p*O_2_) to variations in background extinction rates, we drive an animal physiological model with the environmental outputs from an Earth system model across intervals spanning the past 541 million years. We find that climate and continental configuration combined to make extinction susceptibility an order of magnitude higher during the Early Paleozoic than during the rest of the Phanerozoic, consistent with extinction rates derived from paleontological databases. The high extinction susceptibility arises in the model from the limited geographical range of marine organisms. It stands even when assuming present-day *p*O_2_, suggesting that increasing oxygenation through the Paleozoic is not necessary to explain why extinction rates apparently declined with time.

## Teaser

Climate and continental configuration combined to make Early Paleozoic animals susceptible to extinction.

## INTRODUCTION

The seminal work of Sepkoski *et al.* (*1*, *2*) constituted a milestone in the quantitative reconstruction of marine (invertebrate) biodiversity over the Phanerozoic (past 541 Ma). Subsequently, the development of community paleobiological databases (*3*, *4*), combined with more robust statistical methods to reduce the impact of sampling and preservation biases (*3*, *5*), has led to further refinements in the Phanerozoic biodiversity curve. However, key features of the long-term global biodiversity patterns are robust, particularly the Early Paleozoic (Cambrian and Ordovician) increase in standing biodiversity, the Permian-Triassic drop and Early Mesozoic recovery, with a rise to peak Phanerozoic biodiversity during the Late Mesozoic through Cenozoic (*5*). Many studies have investigated the drivers of these temporal trends but have done so mainly in isolation and focusing on short intervals of time spanning mass extinctions or intense radiation (*6*–*8*). Therefore, attempts to unravel the long-term drivers of biodiversity change throughout the Phanerozoic have been scarce (*5*, *9*–*11*). Those that have done so, such as in a recent numerical biodiversification model study (*11*), have often focused on the net diversification rate. In contrast, the distinct contributions of diversification versus extinction have remained underexplored.

Analyses of the Paleobiology Database (PBDB) reveal that major variations in apparent marine extinction rates have occurred outside of mass extinctions during the Phanerozoic (*2*, *12*, *13*). “Background” extinction rates are particularly elevated during the Early Paleozoic (Cambrian and Ordovician) (*12*, *13*). For this reason, these periods are sometimes considered separately in paleontological analyses (*12*, *14*). For example, it has been proposed that the high Early Paleozoic extinction rates reflected an interval of lower-than-modern atmospheric oxygen concentrations (*p*O_2_) throughout the Cambrian and Ordovician (ca. 0.4 times modern) (*15*), the latter *p*O_2_ estimates aligning with the results of long-term carbon cycle (box) models (*16*–*18*). However, some geochemical proxies suggest that the Early Paleozoic *p*O_2_ may have been closer to modern (*19*). Moreover, Earth system model simulations resolving ocean circulation show that Cambrian and Ordovician continental configurations lead to a poorly ventilated and largely anoxic seafloor—potentially reconciling Early Paleozoic redox proxies for deep-sea anoxia (*16*) with a *p*O_2_ possibly as high as modern. These elements highlight that Early Paleozoic *p*O_2_ remains poorly constrained and might have been closer to modern, inviting us to revisit the cause of elevated Early Paleozoic extinction rates (*15*).

Here, we investigate the evolution of the susceptibility of marine animal background extinction during the Phanerozoic, assuming that ocean temperature and dissolved oxygen concentrations together exert a first-order control on marine habitability (*15*, *20*) and that global environmental perturbation (represented here using global warming) constitutes an essential driver of extinction. We use an ecophysiological model forced by environmental conditions of ocean temperature and ocean oxygenation simulated with an Earth system model. On the basis of previous works, three main factors were identified as exerting a first-order control on oceanic oxygen concentrations, thus potentially on marine biodiversity and extinctions, during the Phanerozoic: the atmospheric oxygen concentration (*p*O_2_) (*15*, *21*), continental configuration (*22*, *23*), and global climatic state (*6*, *23*) (the latter two further affecting ocean temperatures). Successive series of simulations allow us to quantify the contributions of these three factors to changes in background extinction rates during the Phanerozoic.

**Fig. 1.**
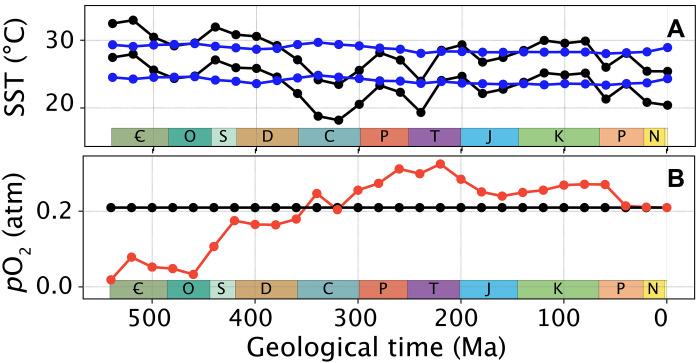
SSTs and atmospheric *p*O_2_ forcing. (**A**) Equatorial (10°S to 10°N) SSTs in the pre- and postwarming states (lower and upper curves, respectively) of the baseline and *p*O_2_ (identical, black lines) and constant SST (blue lines) series of simulations. (**B**) Atmospheric *p*O_2_ in baseline and constant SST (identical, black line) and *p*O_2_ (red line) series of simulations. Ꞓ, Cambrian; O, Ordovician; S, Silurian; D, Devonian; C, Carboniferous; P, Permian; T, Triassic; J, Jurassic; K, Cretaceous; P, Paleogene; N, Neogene.

We start by simulating the potential evolution of global climate and ocean biogeochemistry during the Phanerozoic using the carbon-centric Grid Enabled Integrated Earth system model (cGENIE) (*24*) (Materials and Methods). We conduct simulations at regular time intervals (every 20 million years) during the Phanerozoic and, for each time slice, generate a “cold” and a “warm” climatic state. The warmer state assumes a quadrupling of *p*CO_2_ compared to the cold state (Fig. 1A), leading to a +5°C increase in equatorial sea surface temperature (SST). This amplitude of global warming was chosen to represent the upper limit of rapid climatic changes known from the geological record, known as ‘hyperthermals” (*25*).

Simulated marine environmental conditions are used as input to an ecophysiological model accounting for the combined impacts of temperature and ocean dissolved oxygen ([O_2_]) on ectotherm habitat viability. The model is based on the metabolic index (*20*). A marine region is defined as viable for a population under a given climate as long as dissolved oxygen supplied by the physical environment exceeds the organism’s oxygen demand (Materials and Methods). This model has been developed and validated for the modern ocean (*20*, *26*). It assumes an infinite dispersal capacity of marine organisms (*15*, *20*).

For each of our 28 Phanerozoic time slices, we evaluate the degree of marine extinction occurring in the model in response to a hyperthermal event. To that end, we simulate standing ecophysiotype biodiversity in the cold and warm climatic states simulated in cGENIE and calculate the magnitude of extinction resulting from warming—referred to hereafter as the simulated “susceptibility of extinction.” This quantity, calculated on a single model time slice, is intrinsically very different from an “extinction rate” derived from paleontological data, which is calculated between two subsequent time slices. Therefore, our simulated trends in susceptibility of extinction cannot be compared with data-derived extinction rates at face value but will permit quantifying the contributions of various environmental factors to changes in extinction risk during the Phanerozoic.

**Fig. 2.**
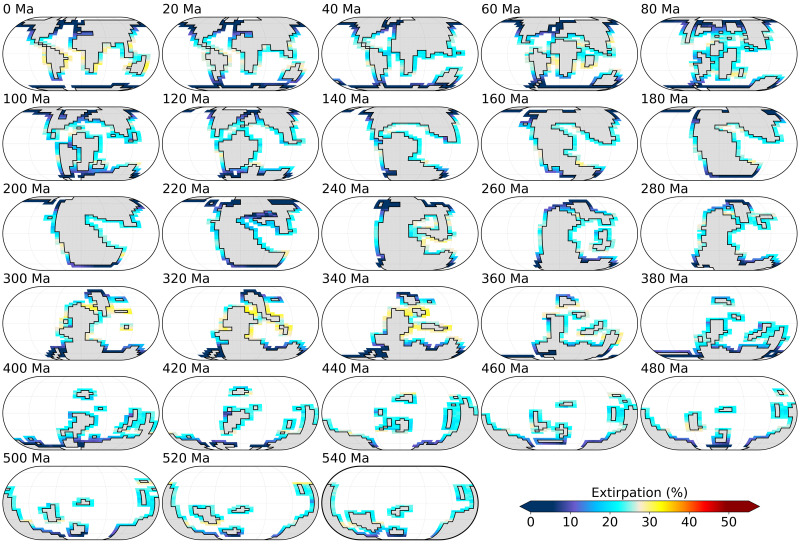
Maps of surface-ocean extirpation rate simulated in response to global warming in the baseline simulations. Extirpation rate is calculated for each grid point as the percentage of ecophysiotypes that are present before global climate warming (i.e., in the cold state) but that are not present in the warm state. Emerged continental masses are shaded gray. Eckert IV projections.

To simulate standing ecophysiotype biodiversity (under the cold and warm climatic states, for each time slice), and in the absence of quantitative constraints on the ecophysiological affinities of ancient marine animals, 1000 physiological ecotypes (herein, ecophysiotypes) are generated, whose physiological characteristics are randomly sampled from probability density functions established on available experimental respirometry data (*15*, *20*). These physiological characteristics consist of three parameters. Each ecophysiotype is first defined by an oxygen demand under resting metabolism conditions (parameter no. 1) and a dependence of this oxygen demand on changes in seawater temperature (parameter no. 2). To constitute viable populations, organisms have to accomplish additional tasks such as reproduction and locomotion, which increases their oxygen demand above resting value. Therefore, each ecophysiotype is also characterized by an increase in oxygen demand necessary for viable populations (parameter no. 3). Extirpation rate is calculated for each model grid point as the percentage of ecophysiotypes that are present in the cold state but that are not present in the warm state (see Fig. 2). In line with previous work (*15*, *27*), we only consider nonpolar shelf environments in our simulations (defined as all nonpolar, upper-ocean model grid cells adjacent to landmasses), because they represent the main part of the Phanerozoic paleontological databases. The same pool of 1000 ecophysiotypes is used for every time slice.

**Fig. 3.**
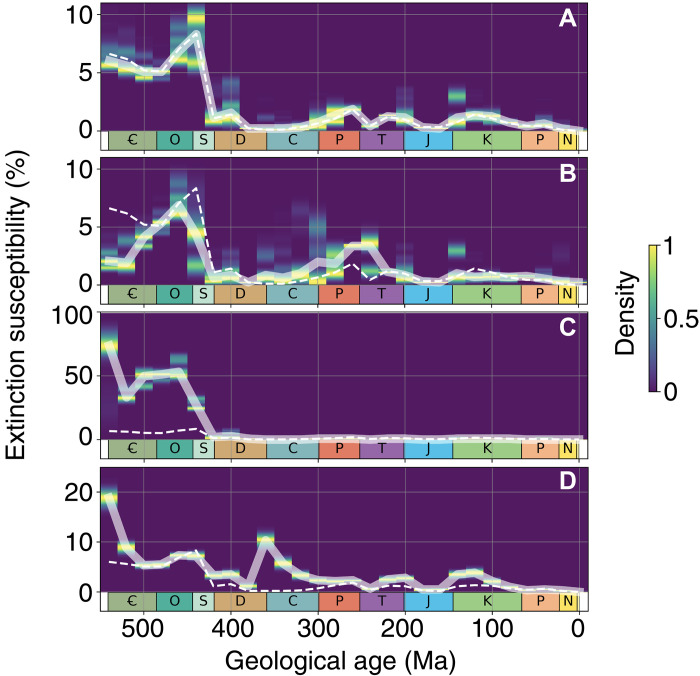
Simulated extinction susceptibility. (**A**) Extinction susceptibility in our baseline simulations with sampling rate fixed at 0.33 (density distribution and thick solid line) and with sampling rate linearly increasing from 0.2 at 540 Ma to 0.8 at 0 Ma (thin dashed line, representing a possibly more complete paleontological sampling toward present day), using 1000 sampling repetitions and sampling prewarming and postwarming states at same shelf grid points. (**B**) Extinction susceptibility in our constant SST simulations with sampling rate fixed at 0.33 (density distribution and solid line, 1000 repetitions using same sampling points). Results of the baseline simulations with sampling rate fixed at 0.33 [1000 repetitions using same sampling points, see (A)] overlaid for comparison (dashed line). (**C**) Same as (B) for *p*O_2_ simulations. (**D**) Same as (A) but using PBDB-derived, collection-based sampling rates. Results of the baseline simulations with sampling rate fixed at 0.33 [1000 repetitions using same sampling points, see (A)] overlaid for comparison (dashed line). Y scale differs in the different panels.

Then, we need to derive a global susceptibility of extinction based on these spatial data. To permit a more robust comparison of our numerical results with temporal trends in marine extinction derived from paleontological data, we explore the impact of incomplete geological sampling in our model using a subsampling approach. Instead of reading model results at face value, we consider that the information in the paleontological databases is incomplete. Hence, we only record a fraction of all model shelf grid points. In other words, for each time slice, we subsample a fraction of all shelf grid points to determine the ecophysiotypes present in the cool and warm states and calculate a global susceptibility of extinction occurring in response to global warming (defined as the percentage of ecophysiotypes that are present in the cold state, which are not present in the warm state). We repeat this procedure 1000 times to calculate uncertainty estimates. Because the fraction of shelf environments documented through geological time is poorly constrained, we arbitrarily set the subsampling rate to 33% in our main simulations. We test alternative subsampling rates (and numbers of repetitions) in our sensitivity analyses and show that varying these parameters does not affect our conclusions. Then, we estimate the resulting probability density function of simulated susceptibility of extinction using a kernel density estimator to quantify uncertainty in our simulations (shading in Fig. 3, A to C). Last, our central estimate for the temporal trend in simulated Phanerozoic extinction susceptibility is obtained by connecting the median values for all time slices (thick lines in Fig. 3, A to C). This subsampling approach avoids giving too much weight to species found in only a few model grid points, which would probably not be sampled and thus not be documented in paleontological databases. Our simple experimental setup featuring a uniform magnitude of global warming through time was not designed to investigate the magnitude of specific ancient extinction events but only the general temporal trends in susceptibility to an idealized warming-driven extinction. This approach is designed to provide a directional comparison in extinction susceptibility, and absolute numbers should not be compared with paleontological databases at face value.

## RESULTS

### Simulated extinction susceptibility

In our first series of "baseline" (best-guess) simulations, in addition to varying the continental configuration, we also vary the atmospheric CO_2_ concentration during the Phanerozoic based on a combination of carbon cycle models and proxy data compilations (*25*, *28*). The resulting global temperature curve simulated in cGENIE (black lines in Fig. 1A) exhibits temporal trends that align well with other climate models (*29*) and temperature proxy data (*30*, *31*), including a warm Early Paleozoic (Cambrian-Devonian), a cooler Late Paleozoic (Carboniferous-Permian) coincident with the Late Paleozoic Ice Age (*32*), a warm (but cooler than the Early Paleozoic) Mesozoic (Triassic-Cretaceous), and a long-term Cenozoic cooling. In these simulations (Fig. 3A), we account for combined changes in continental configuration and global climate but consider a modern atmospheric *p*O_2_ (black line in Fig. 1B). Simulated extinction susceptibility exhibits a sudden drop from an Early Paleozoic mean of 6.2% (SD, 1.2%) from the Cambrian to Ordovician (540 to 440 Ma)—meaning that ~6 of 100 model species are driven extinct following global climate warming—to much lower values during the rest of the Phanerozoic, with a mean of 0.8% (SD, 0.5%). These changes represent an eightfold decrease in mean extinction susceptibility following the Ordovician. Sensitivity analyses reveal that simulated temporal trends are robust when model parameters are varied. That includes varying the initial random sampling of the physiological characteristics of the model ecophysiotypes (fig. S1), the model ecophysiotype pool size (between 100 and 10,000 ecophysiotypes, compared to 1000 in our standard simulations; fig. S2), the random sampling protocol (fig. S3), and random sampling rate used to represent incomplete geological sampling (between 0.1 and 0.75, compared to 0.33 in our standard simulations; fig. S4; or increasing through time, Fig. 3A), the model spatial domain (figs. S5 and S6), and assumptions regarding ocean phosphate inventories (fig. S7). Sensitivity tests for the spatial domain, in particular, demonstrate that our results are not overly dependent on the (simplified) representation of shelf environments (defined in our model as all cells adjacent to landmasses) (figs. S5 and S6).

**Fig. 4.**
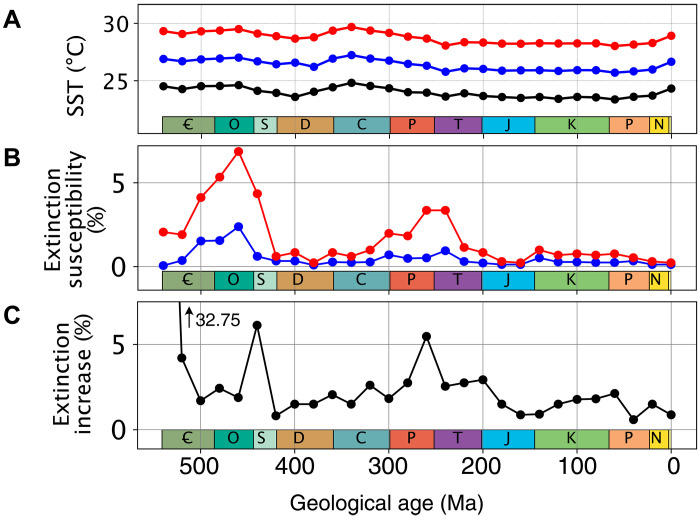
Sensitivity of simulated extinction risk to the magnitude of global warming. (**A**) Sea surface temperature in the prewarming state of the constant SST series of simulations (black line), for a *p*CO_2_ doubling (blue line) relative to the prewarming state and for a quadrupling (red line). Black and red curves identical to Fig. 1A (blue curves). (**B**) Extinction susceptibility in the constant SST simulations with sampling rate fixed at 0.33 (1000 repetitions using same sampling points), when considering a doubling (blue line) or quadrupling (red line) of *p*CO_2_ [i.e., shifting from respectively black to blue or black to red in (A)]. Red line similar to Fig. 3B. (**C**) Sensitivity of simulated extinction susceptibility to the magnitude of global warming, i.e., increase in extinction susceptibility simulated when increasing the magnitude of global warming from a doubling to a quadrupling of *p*CO_2_ (i.e., when increasing equatorial SST rise from ~2.5° to ~5°C).

To disentangle the contributions of changes in the global climate state and continental configuration to the simulated extinction trend through the Phanerozoic, we conduct an additional series of simulations under a constant global climatic state (blue curves in Fig. 1A). Similar to (*22*), climatic detrending is achieved by varying *p*CO_2_ in the model so that the equatorial SST of every time slice approximates the median equatorial SST in the baseline simulations (ca. 24.5°C before warming). This second series of simulations is referred to as “constant SST” hereafter. Similar to the baseline simulations, atmospheric *p*O_2_ is set to modern. In these constant SST simulations, only the continental configuration is thus varied through time. The consequence of a constant-through-time climate state is that particularly high extinction susceptibility now occurs in the Early Paleozoic (Late Cambrian and Ordovician) and during the Permian-Triassic transition (Fig. 3B). Comparing the baseline and constant SST (Fig. 3, A and B) series of experiments reveals the role of continental configuration versus climate in driving trends in extinction susceptibility in our model. From this, we deduce that the continental configuration of the Permian-Triassic transition favors high extinction susceptibilities but also that the cooler climatic conditions act to reduce extinction susceptibility in our baseline simulations (Fig. 1A). Note that the short-term global warming at the Permian-Triassic boundary is not resolved at the prescribed 20–million year temporal model resolution (*33*). The opposite is observed for the Early Cambrian, when the continental configuration is not particularly favorable to extinction, but the warm climate elevates extinction risk. Sensitivity tests show that these temporal trends in extinction susceptibility are largely independent of the magnitude of warming perturbation assumed (~2.5°C versus ~5°C) (Fig. 4). However, three time periods do stand out as being particularly sensitive to the magnitude of the environmental perturbation: the Early Cambrian, the Ordovician-Silurian transition, and the Permian-Triassic transition. Unfortunately, the current formulation of the metabolic index cannot be used to specifically evaluate the contribution of global climate change to the Late Ordovician Mass Extinction, which happened in response to global cooling rather than warming (*6*, *34*).

In a third and final series of experiments (named “*p*O_2_”), we quantify the combined impacts of changes in the continental configuration and global climate (as per baseline) with the additional assumption of changing atmospheric *p*O_2_ through time. For this, *p*O_2_ estimates are taken from the recent update of the GEOCARBSULF model of (*28*) (red line in Fig. 1B). The result of this analysis is consistent with the findings of (*15*). Low Early Paleozoic *p*O_2_, by reducing the thermal safety margins of marine ectotherms facing global climate warming, increases extinction risk by an order of magnitude during the Cambrian and Ordovician (compare Fig. 3, A and C; note the different *y* axes).

### Drivers of Phanerozoic extinction rates

The unexpected result from our coupled global marine environmental and ecophysiological modelling is that the extinction susceptibility simulated in response to global warming is substantially higher in the Cambrian and Ordovician than in more recent time slices, even if we assume that atmospheric *p*O_2_ throughout the Phanerozoic was the same as modern (Fig. 3A). In addition, the high Early Paleozoic global extinction susceptibilities are not associated with high local extirpation rates (Fig. 2). Extirpation rates for more recent periods (e.g., 0 Ma or 300 to 340 Ma) are higher than those for 460 to 540 Ma, suggesting that more complex mechanisms modulate extinction susceptibility in the model, which we explore below.

**Fig. 5.**
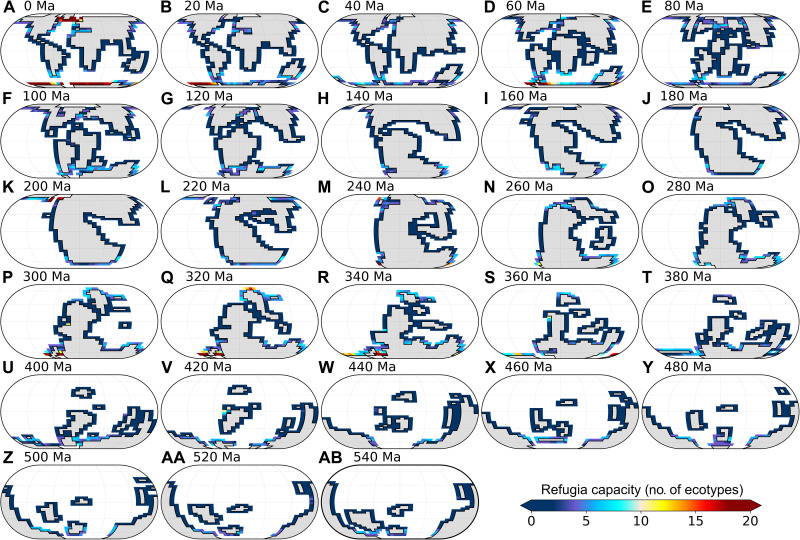
Maps of surface-ocean simulated refugia capacity (expressed as a number of ecophysiotypes) in the baseline simulations. Refugia capacity is calculated in each grid point as the number of ecophysiotypes (present in the cold state) that were not present in this specific grid point in the prewarming state but are present in the postwarming state. Emerged continental masses are shaded gray. Eckert IV projections.

Under certain conditions, ecophysiotypes whose ecophysiological requirements are not fulfilled in the low latitudes after global warming can migrate poleward and occupy habitats at higher latitudes (and thus do not become extinct). These high-latitude habitats constitute refugia for organisms facing global environmental disturbances (*35*). Figure 5 shows that high low-latitude extirpation rates at 0 Ma or 300 to 340 Ma in the baseline simulations are (at least in part) counterbalanced by the development of refugia at higher latitudes. The development of these high-latitude refugia is associated with local increases in the capacity of the environment to sustain a high metabolism (i.e., metabolic index, fig. S8), resulting from the combination of a substantial increase in ocean [O_2_] (fig. S9) and muted SST rise (fig. S10). These unexpected local climatic signals arising in response to global warming are due to the partial melt (and persistence of a fraction) of the local sea-ice cover (figs. S11 to S13), which favors ocean-atmosphere O_2_ transfers (due to sea-ice partial melt) while reducing SST rise (due to sea-ice persistence). This muted SST change is important in both maintaining metabolic oxygen demand at the same level and preventing any warming-induced limitation of O_2_ dissolution in seawater (*36*). The refugia developing in more recent periods (e.g., 0 or 300 to 340 Ma) lower the susceptibility of extinction for these time slices. In contrast, the Early Paleozoic is comparatively more prone to extinction in our model.

**Fig. 6.**
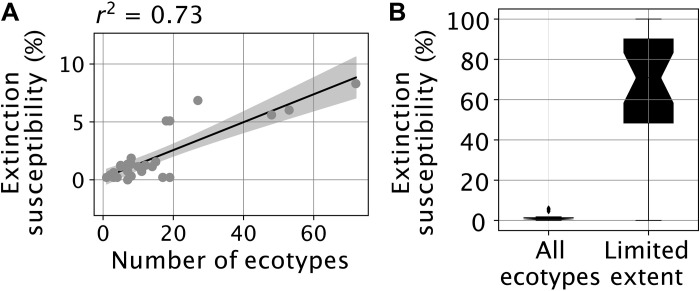
Extinction susceptibility and ecophysiotype geographical range size in the baseline simulations. (**A**) Linear correlation between simulated extinction susceptibility (median value calculated by sampling 1000 times at same locations; thick line in Fig. 3A) and number of ecophysiotypes with limited spatial extent (<10 equal-area model shelfal grid cells at any depth level in the prewarming state). Points represent each of the 28 time slices and the line is the linear correlation line (with 95% confidence interval), the coefficient of which is provided on top of the panel. (**B**) Extinction susceptibility for ecophysiotypes with limited spatial extent present in the prewarming state (<10 equal-area shelfal model grid cells at any depth level) versus for all ecophysiotypes. Boxplots were calculated on the basis of the individual extinction susceptibilities calculated for each of the 28 time slices studied without accounting for uncertainties in spatial sampling. Results for other series of experiments are provided in figs. S15 and S16.

The differential refugia capacity, however, does not explain the order-of-magnitude difference in extinction susceptibility during the Early Paleozoic versus that of some more recent periods (e.g., 140 to 180 Ma). Nor does it explain the step change in extinction risk simulated between 440 and 420 Ma in the baseline simulations (or between 460 and 440 Ma in the constant SST experiments) (Figs. 3 and 5). At the Phanerozoic timescale, extinction susceptibility positively correlates in the model with the number of ecophysiotypes having a limited geographical spatial range (Fig. 6A), the latter ecophysiotypes effectively displaying an extinction susceptibility substantially higher than ecophysiotypes occupying a large geographical space (Fig. 6B) (see also figs. S14 to S16). Therefore, the simulated high Early Paleozoic extinction susceptibility results from the existence of many ecophysiotypes with a limited geographical range in the prewarming state, which are preferentially driven extinct in response to global warming.

Why, then, are there so many ecophysiotypes with limited extent in these oldest model time slices? One possibility is that the Early Paleozoic permits stabilizing model ecophysiotypes with unusual ecophysiological characteristics, whose requirements are fulfilled in a small ocean region only, and that these ecophysiotypes are not found in other time slices. However, ecophysiotypes with a limited extent in the Early Paleozoic are equally present in other time slices, only showing a larger (and monotonically increasing) spatial cover toward the modern (fig. S17). An alternative hypothesis, and the one we prefer, is that the Early Paleozoic high-latitude marine environment is spatially highly heterogeneous, leading to environmental fragmentation. Spatial variations in physical ocean parameters ([O_2_] and temperature) create a very variable ecophysiological landscape in the southern high latitudes (fig. S18). As a consequence of this and the lack of northern high-latitude continental shelves in the Early Paleozoic, many ecophysiotypes are present in just a few model grid points in the prewarming state (fig. S19); their ecological niche disappears in response to global warming, and they are consequently driven extinct (Fig. 6).

### Sampling structure and biases in the PBDB

To quantify the likely impact of heterogeneous preservation and sampling of the fossil record [e.g., (*37*)] on our simulated extinction trend, we calculated new extinction susceptibilities by sampling the maps of simulated ecophysiotypes based on the number of PBDB collections documented for each cGENIE grid point (Materials and Methods). While the subsampling approach previously used in the standard model assumes an incomplete but spatially uniform (random) sampling bias, this alternative approach accounts for the spatially heterogeneous nature of the paleontological sampling biases, with most data coming from North America and Europe (*37*) (fig. S20). Figure 3D shows that PBDB-derived, collection-based subsampling leads to higher extinction susceptibility during virtually the whole Phanerozoic—an expected result from the relative undersampling of the high paleolatitudes in the PBDB (fig. S20), which are refugia for ecophysiotypes facing global warming in our simulations. While main temporal trends still stand, extinction susceptibility displays drastic increases during the earliest Cambrian and the Devonian-Carboniferous transition suggesting a potential sampling factor in the high reconstructed extinction rates during these time intervals. An alternative subsampling method based on the number of PBDB entries (instead of collections) per cGENIE grid point gives similar results (fig. S21).

## DISCUSSION

Simplifications in our numerical modeling (which represents a susceptibility of extinction in response to climatic perturbations) and biases of the PBDB (*12*) prohibit direct numerical comparison of our models and data. However, qualitative comparison of the temporal trends is instructive and reveals that it is not necessary to assume that atmospheric *p*O_2_ was low during the Early Paleozoic to obtain a simulated extinction susceptibility that is substantially higher during the Cambrian and Ordovician than during the rest of the Phanerozoic (Fig. 3). This does not necessarily imply that Early Paleozoic atmospheric *p*O_2_ was as high as modern but does indicate that oxygen was not the only important factor. While *p*O_2_ has a first-order impact on simulated extinction susceptibility (Fig. 3C) in line with previous studies (*15*), we find that climate and continental configuration acted synergistically to make Early Paleozoic marine ecosystems particularly susceptible to elevated extinction and lower atmospheric oxygen need not be invoked.

In our model, ecophysiotypes with a small geographical range size display a higher extinction risk in response to global warming (Fig. 6), in line with previous analyses of the predictors of extinction risk based on the PBDB (*14*, *38*). This mechanism, combined with the highly heterogeneous ecophysiological landscape resulting from the Early Paleozoic continental configuration (and climate) (fig. S18), explains the high extinction susceptibility reconstructed for the Cambrian and Ordovician. Our coupled climate-ecophysiology model also suggests that global climate state and continental configuration exert control on marine extinction susceptibility at the Phanerozoic timescale. As illustrated in our baseline simulations, extinction proceeds differently in greenhouse and icehouse climates. In our coldest time slices (0 to 20 Ma and 260 to 360 Ma; to a lesser extent in the 60-Ma time slice; see Fig. 1A), high low-latitude extirpation is, in part, counterbalanced by the development of refugia at higher latitudes, where species migrating poleward can survive following global warming (Figs. 2 and 5). The development of high-latitude refugia requires the (only) partial melt of sea ice. In our simulations, this mechanism occurs in the modern glacial state and during the Permian-Carboniferous glaciation. It does not occur in warmer climates. We note, however, that the relative resilience to warming-induced extinctions in (modern-like) cool environments may partly arise from the fact that modern organisms that are used as the basis for the ecophysiotypes have adapted to these conditions. This possible contribution cannot be discarded, and it will be important to quantify it in future work.

The second period identified as the most prone to leading to extinction in our series of constant SST simulations (featuring a roughly constant global climatic state) is the Permian-Triassic transition, considered as the largest mass extinction over Earth’s history (*1*, *39*) (Fig. 3B). Simulations accounting for PBDB-derived sampling biases (Fig. 3D) demonstrate that incomplete geological sampling leads to an overestimation of the simulated susceptibility of extinction. Extinction rates documented during the earliest Cambrian and latest Devonian may thus be substantially overestimated in the PBDB, with implications for the role sampling may play in our reconstruction of major extinction intervals through time (*40*).

Numerical approaches such as those presented here provide an important tool for exploring the coevolution of global climate and the marine biosphere at the Phanerozoic timescale and additionally provide a promising approach to bridging the gap between model outputs and the geological record (here, the PBDB). Nevertheless, further refinements are needed. A first limitation is that global warming was used in our model to destabilize ecological niches and derive a susceptibility of extinction through time, while background extinctions are not necessarily driven by global warming during the Phanerozoic. Another limitation is that our model implicitly considers that the dispersal capacity of the model ecophysiotypes is infinite, as is the carrying capacity of marine habitats (*11*). No factor other than temperature and dissolved oxygen concentrations limits the extent of model ecophysiotypes, which systematically occupy their whole ecological niches. Implementing migration in our model would permit investigating the impact of physical barriers and the kinetics of global climate change. It would also permit accounting for the contribution of (seasonal to centennial) climatic variability, such as simulated by recent global climate models of, e.g., the Coupled Model Intercomparison Project (*41*), on ecological niche stability and marine extinction susceptibilities. Noteworthily, accounting for dispersal limitation would lead to higher simulated extinction susceptibility but would probably not alter our conclusions. Our model results are, therefore, likely a conservative estimate of extinction susceptibility. Previous work demonstrated that the Early Paleozoic continental configuration, due to the limited latitudinal continuity of landmasses, makes organisms facing global climate change particularly vulnerable (*27*). In addition, earliest planktotrophic larvae likely appeared at (or very close to) the base of the Ordovician (*42*, *43*), suggesting that Early Paleozoic (and especially Cambrian) marine animals were limited in their dispersal abilities compared to later animals. Therefore, a finite dispersal capacity of model ecophysiotypes might make the post-Ordovician drop in extinction susceptibility even more pronounced. Finer model resolution would also be an obvious advantage in being able to better account for the diversity of environmental niches but equally creates its own computational challenges if dissolved oxygen concentrations are to be simulated globally and to steady state, and for multiple time intervals through the Phanerozoic.

Another future direction relates to the representation of the marine biosphere. In the current ecophysiological model version, previously validated for the modern (*26*) and successfully applied to the geological past (*20*), metabolic rates of most ecophysiotypes monotonically increase with temperature, leading to a monotonic decrease in ecophysiotype fitness. However, empirical results (*44*, *45*) and models (*46*) demonstrate that natural species thermal performance curves are unimodal and metabolic rates decline rapidly once the optimal temperature is exceeded. Assembling a database to represent this increase in ecophysiotypes fitness with increasing temperatures will permit capturing more finely the latitudinal diversity gradient (*47*–*49*) and will thus offer a better representation of marine biodiversity. It should also be noted that our approach is rooted in the modern and that organisms that populated deep-time oceans may have had different environmental affinities. Although the analysis of experimentally derived estimates of thermal tolerance limits of >2000 terrestrial and aquatic species suggests that the upper thermal limits of metazoans have not changed much throughout the Phanerozoic (*50*), it has also been suggested that the Paleozoic fauna may have been characterized by lower rates of metabolism (*51*). It would also be informative to test the impact of implementing a representation of the legacy of past extinctions in defining the ecophysiotypes present in the next time slice (whereas the same pool of ecophysiotypes is considered in every time slice in the current model). Last, it might also be worth representing ecophysiotype adaptation and evolution in response to climate change (through time-evolving ecological niches) (*52*). However, such model development would probably not drastically affect our conclusions because of the rapidity of the climatic perturbations considered here (hyperthermals).

Overall, our coupled climate-ecophysiology model illustrates how continental configuration and climate state specific to the Early Paleozoic render metazoans particularly prone to extinction. Although our results reaffirm the possible contribution of a reduced *p*O_2_ to increasing Early Paleozoic extinction rates (*15*, *16*), they also reconcile the vision that extinction susceptibility was much higher during the Cambrian and Ordovician than during the rest of the Phanerozoic with a relatively constant atmospheric *p*O_2_ through time [possibly as high as modern (*22*)]. Our simulations further suggest that the continental configuration may have also played a key role in setting the conditions for the largest Phanerozoic mass extinction at the Permian-Triassic boundary. Last, PBDB-based subsampling of our model output reveals that extinction rates documented during the latest Devonian may be substantially overestimated in the PBDB.

## MATERIALS AND METHODS

### Earth system model simulations

#### Description of the model

cGENIE (*24*) is an Earth system model of intermediate complexity. It is based around a three-dimensional ocean circulation model coupled to a two-dimensional (2D) energy-moisture-balance atmospheric model. The model was configured on a 36 × 36 equal-area grid with 17 unevenly spaced vertical levels to a maximum 5890-m depth in the ocean. The cycling of carbon and associated tracers in the ocean is based on a single (phosphate) nutrient limitation of biological productivity (*6*) but adopts the Arrhenius-type temperature-dependent scheme for the remineralization of organic matter exported to the ocean interior of (*53*). Despite its low spatial resolution, cGENIE has been shown to satisfactorily simulate first-order ocean [O_2_] spatial patterns and values in the modern (*24*) and geological past (*6*, *7*).

#### Description of the numerical experiments

We adopted the (flat-bottomed) Phanerozoic continental reconstructions of (*54*, *55*) but substituted the deep-ocean bathymetry of (*56*) when available (140 to 0 Ma) to account for mid-ocean ridges, following previous work (*22*). Solar luminosity was adapted for each time slice after Gough (*57*). We used a null eccentricity-minimum obliquity orbital configuration, which provides an equal mean annual insolation to both hemispheres with minimum seasonal contrasts. Atmospheric CO_2_ concentration was varied in our baseline experiments after (*25*), when available (≤400 Ma), and (*28*) for deeper time slices. In detail, we ran two series of cGENIE simulations for our baseline experiments, to generate the prewarming and postwarming global climatic states, by multiplying the *p*CO_2_ values of (*25*) and (*28*) (see above) by 0.5 and 2.0, respectively. These multiplication factors were chosen to provide a quadrupling of *p*CO_2_ (permitting to simulate the +5°C low-latitude warming required for our ecophysiological simulations; see main text), while staying as close as possible to the “target” values of (*25*) and (*28*). Specifically, the simulated low-latitude (10°S to 10°N) SST warming amounts to +4.80°C (SD, 0.21°C), or equivalently a mean global SST increase of +4.77°C (SD, 0.23°C). We note that atmospheric *p*CO_2_ during the Devonian may have been lower than considered in our simulations, which would lead to a colder Devonian climate at 420 and 400 Ma (*18*). We also conducted additional simulations (constant SST experiments), in which we varied *p*CO_2_ so as to approximatively correct for the global climatic trend and therefore leave equatorial SST mainly invariant. Atmospheric oxygen concentrations were set to modern (20.95%) in our baseline and constant SST simulations, but varied according to (*28*) in our *p*O_2_ experiments (Fig. 1B). Ocean nutrient inventory was kept invariant to modern (2.1 μmol kg^−1^ PO_4_) in our experiments (only varied for the purpose of sensitivity testing).

To generate the physical atmospheric boundary conditions required by cGENIE for each different cGENIE continental configuration, we ran experiments using the slab version of the Fast Ocean Atmosphere Model (FOAM) (*58*) for 100 years (until equilibrium). This setup of the FOAM model couples an atmospheric general circulation model to a 50-m “slab” mixed-layer ocean of resolution 1.4° × 2.8° (latitude × longitude) (*59*, *60*). We then derived the 2D wind speed and wind stress, and 1D zonally averaged albedo forcing fields required by the cGENIE model, using the “muffingen” open-source software (DOI: 10.5281/zenodo.7545809), following the methods used in (*6*, *22*, *61*).

cGENIE simulations were initialized with a sea-ice free ocean and homogeneous temperature and salinity in the ocean (5°C and 33.9‰, respectively) and integrated for a total of 8000 years (a duration largely sufficient to reach ocean thermal equilibrium and upper-ocean dissolved oxygen equilibrium).

### Ecophysiological modeling

We adapted the probabilistic ecophysiological model of extinction vulnerability of (*15*), which is based in turn on the metabolic index developed and validated in (*26*) and (*20*). Metabolic habitat viability is calculated following Eq. 1

$$\text{Metabolic habitat viability}=\sum_{\min(A_{o},E_{o},\phi_{\mathrm{crit}})}^{\max(A_{o},E_{o},\phi_{\mathrm{crit}})}\phi >\phi_{\mathrm{crit}}$$ (1)

with

$$\phi =A_{o}\;\frac{pO_{2}}{\exp\left[\frac{-E_{o}}{k_{B}}\left(\frac{1}{T}-\frac{1}{T_{\mathrm{ref}}}\right)\right]}$$ (2)

ϕ is the metabolic index defined following (*20*) and (*15*). Metabolic habitat viability defines the fraction of model ecophysiotypes that can live in the oceanic region investigated. Ocean temperature *T* and seawater *p*O_2_ are taken from our Earth system model simulations. *k*_B_ is the Boltzmann constant. *T*_ref_ is a reference temperature of 15°C. At the individual organism scale, *A*_o_ is the inverse of the hypoxic threshold of the organism (the minimum required seawater *p*O_2_ to sustain resting aerobic metabolism), *E*_o_ is the temperature dependency of the hypoxic threshold, and ϕ_crit_ is the multiplicative increase in oxygen supply that is required to support ecologically sustainable populations. Following (*15*), values for *A*_o_, *E*_o_, and ϕ_crit_ are randomly sampled for each ecophysiotype from probability density functions established on laboratory experiments and the observation of species distribution (*20*).

In our standard model simulations, following (*15*), we generate 1000 ecophysiotypes and consider nonpolar shelf environments only, defined as all nonpolar model grid cells adjacent to landmasses in the upper three cGENIE ocean levels, down to a depth of ca. 285 m (but see sensitivity tests for additional experiments using alternative numbers of ecophysiotypes and considering other oceanic regions; figs. S2, S5, and S6).

We calculate extinction susceptibility as the loss of ecophysiotypes in response to a +5°C equatorial warming (*15*). Global climate change is simulated in cGENIE using a quadrupling of atmospheric *p*CO_2_ (see previous section) and is intended to represent a hyperthermal event of the same order of magnitude as the Paleocene-Eocene Thermal Maximum (*62*). We also conduct a sensitivity test with a +2.5°C equatorial warming.

We extend the original model of (*15*) through an explicit representation of incomplete geological sampling bias. This process modifies how a global extinction susceptibility is derived from spatially resolved maps of metabolic habitat viability. Instead of calculating extinction susceptibility at face value based on all ecophysiotypes present in the prewarming and postwarming states, we subsample shelf grid points to account for incomplete geological data sampling. In detail, we extract 33% of all equal-area model grid points and calculate the extinction susceptibility based on the ecophysiotypes found in these grid cells only, and repeat this approach 1000 times for each of our 28 time slices. The result is, for each time slice, a probability density function of simulated extinction susceptibility, estimated using a kernel density estimator. Subsampling ensures that ecophysiotypes present in few model grid cells only would not affect too strongly the calculation of global extinction susceptibilities. This approach is motivated by the fact that such ecophysiotypes would probably not be documented in the paleontological databases. It also ensures that our results are not overly dependent on the environmental conditions simulated in a few cGENIE model grid points but rather represent large-scale environmental patterns. Last, we determine the most probable temporal evolution of Phanerozoic global model extinction susceptibility by joining the median extinction susceptibilities derived for each time slice from the probability density function. In our standard simulations, we subsample the prewarming and postwarming habitat viability maps at the same locations. A sensitivity analysis to random sampling approach (subsampling the prewarming and postwarming habitat viability maps at different locations; fig. S3) and rate (fig. S4) are provided as Supplementary Materials.

### Paleontological data

#### Downloading fossil data

Fossil occurrence data of all marine metazoans were downloaded from the PBDB on 22 February 2022. We restricted downloads to regular taxa (“Preservation = regular taxa only”). Occurrences with uncertain genus or species attribution were excluded (“Modifiers = exclude uncertain gen. and sp.”). Downloaded data were restricted to marine environment (“Environment = any marine, carbonate, siliciclastic”). A total of 886,252 marine metazoan fossil occurrences were downloaded.

In keeping with previous studies (*15*, *63*), fossil data of the following classes were omitted: Ostracoda, Arachnida, Insecta, Reptilia, and Mammalia. In detail, Ostracoda were excluded because the poor database quality, combined with the high diversity of this group, may induce important biases (*63*, *64*). Arachnida and Insecta are terrestrial and documented in marine sediments only under very specific conditions (*63*). Reptilia were excluded because they are either terrestrial or air breathing (*15*). Mammalia were excluded because they are endotherms, while the metabolic index applies to ectotherms. Lagerstätten were also excluded. We also excluded occurrences with unknown paleocoordinates and with age older than 550 Ma. After applying these filtering criteria, 741,860 fossil occurrences of 30,387 marine metazoan genera were used in this work.

#### Calculating paleocoordinates

Paleocoordinates of individual fossil occurrences were calculated on the basis of present-day longitude-latitude coordinates and geological age (both available in downloaded PBDB data), using pyGPlates and the rotational model of Scotese and Wright (*54*). For each occurrence belonging to a given time bin, the closest oceanic grid point was found in the cGENIE simulation of corresponding age, provided that the identified closest oceanic grid point was no further than 2000 km (the PBDB occurrence being otherwise discarded; fig. S20).

#### PBDB-derived sampling and extinction rates

In an effort to represent the impact of heterogeneous geological sampling, we derived sampling rates from the number of collections found in our cured PBDB data (and also conducted a sensitivity test using the number of PBDB entries). For each time slice, we built a PBDB-derived sampling rate map by (i) calculating the paleocoordinates of each PBDB entry included in the time bin and identifying the corresponding cGENIE grid cell, (ii) extracting the number of unique collections found in each cGENIE grid cell, (iii) converting the number of collections into a sampling rate, assuming that sampling rate linearly increases from 0 (in grid points with 0 collections) to 1 (in grid points with a number of collections greater or equal to the 95th percentile of the distribution of the number of collections per cGENIE grid points in cGENIE grid points having at least 1 collection, calculated over all time slices). Resulting maps are shown for each time slice in fig. S20.

We calculated extinction susceptibility by sampling the ecophysiotypes living in the cold and warm climatic states using the sampling rate maps. In each cGENIE grid cell, we randomly extracted a given number of possible ecophysiotypes, varying from 0 (if sampling rate = 0) to the total number of ecophysiotypes considered in the model (1000 in the standard simulations; if sampling rate = 1). It should be noted that while we subsample these model ecophysiotypes, all subsampled ecophysiotypes will not be viable in each cGENIE grid cell, and that the same randomly generated subset of possible ecophysiotypes is used to subsample the cold and warm climatic states. We calculated an extinction susceptibility in response to global climate warming based on the ecophysiotypes extracted in the cold and warm simulations. We repeated the random extraction 1000 times to obtain a probability density function of the simulated susceptibility of extinction.
