# Supplementary material for: Why the Early Paleozoic was intrinsically prone to marine extinction
Source: Sci Adv. 2023 Aug 30;9(35):eadg7679. doi: 10.1126/sciadv.adg7679 (PMC10468122; doi:10.1126/sciadv.adg7679)
Supplement: Supplementary file 1 — Figs. S1 to S21 [file sciadv.adg7679_sm.pdf]

Science Advances
AAAS

Supplementary Materials for

Why the Early Paleozoic was intrinsically prone to marine extinction

Alexandre Pohl *et al.*

Corresponding author: Alexandre Pohl, alexandre.pohl@u-bourgogne.fr

Sci. Adv. **9**, eadg7679 (2023)
DOI: 10.1126/sciadv.adg7679

This PDF file includes:

Figs. S1 to S21

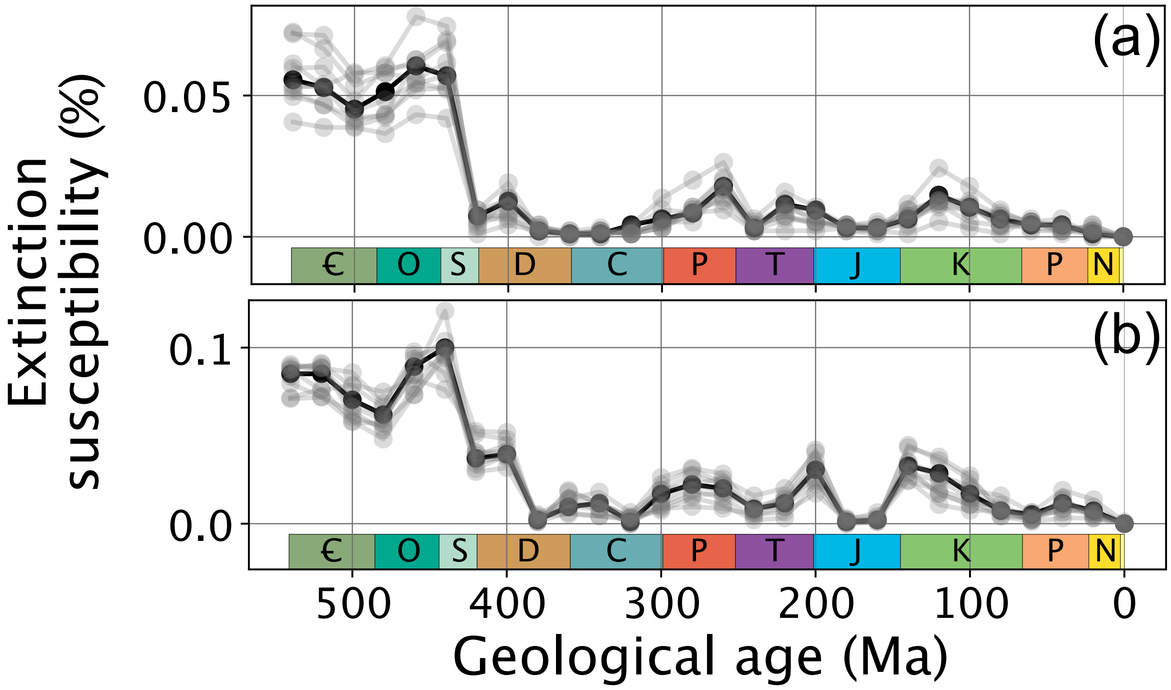

Fig. S1.

Sensitivity test to initial ecophysiotype sampling pool. Simulated extinction susceptibility during the Phanerozoic in the 'baseline' simulations (solid black line) and using 10 alternative randomly-sampled initial ecophysiotype pools (grey lines), discarding either (a) no ecophysiotypes or (b) ecophysiotypes occupying less than 10 (equal-area) model grid cells. €: Cambrian, O: Ordovician, S: Silurian, D: Devonian, C: Carboniferous, P: Permian, T: Triassic, J: Jurassic, K: Cretaceous, P: Paleogene, N: Neogene.

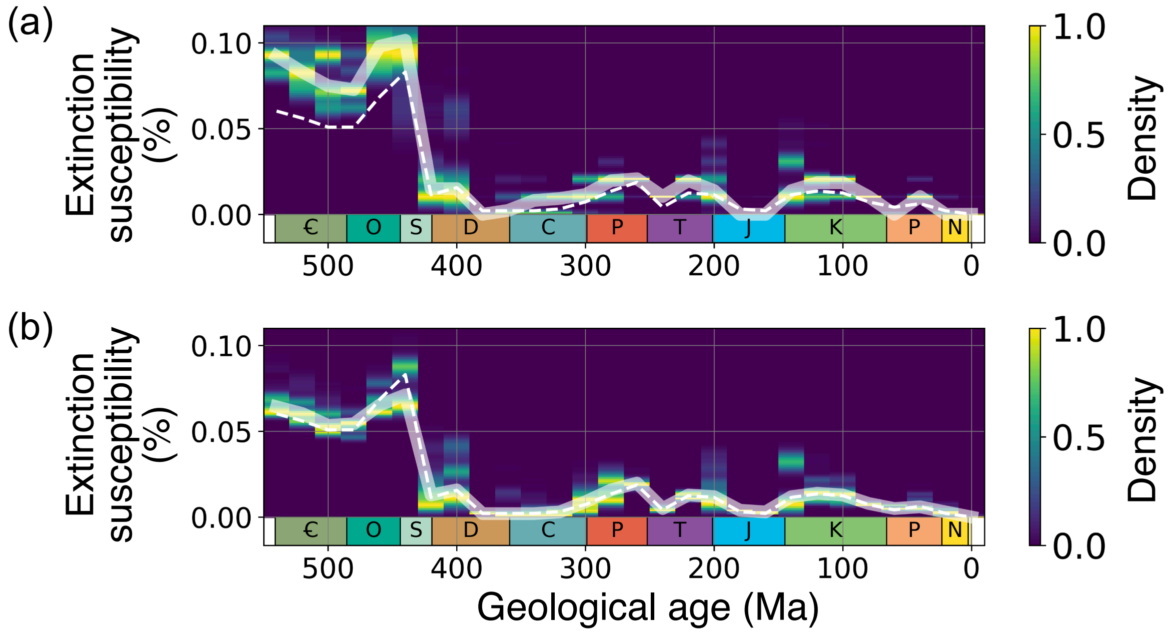

Fig. S2.

Sensitivity test to initial number of ecophysiotypes. Simulated extinction susceptibility during the Phanerozoic. (a) 'Baseline' simulations using 100 (density distribution and thick solid line) and 1000 ecophysiotypes (thin dashed line, like Fig. 3A). (b) 'Baseline' simulations using 10,000 (density distribution and thick solid line) and 1000 ecophysiotypes (thin dashed line, like Fig. 3A). All simulations use a sampling rate of 0.33, 1000 sampling repetitions and sample pre-warming and post-warming states at same shelf grid points. Same as Fig. 3A but using alternative number of ecophysiotypes. €: Cambrian, O: Ordovician, S: Silurian, D: Devonian, C: Carboniferous, P: Permian, T: Triassic, J: Jurassic, K: Cretaceous, P: Paleogene, N: Neogene.

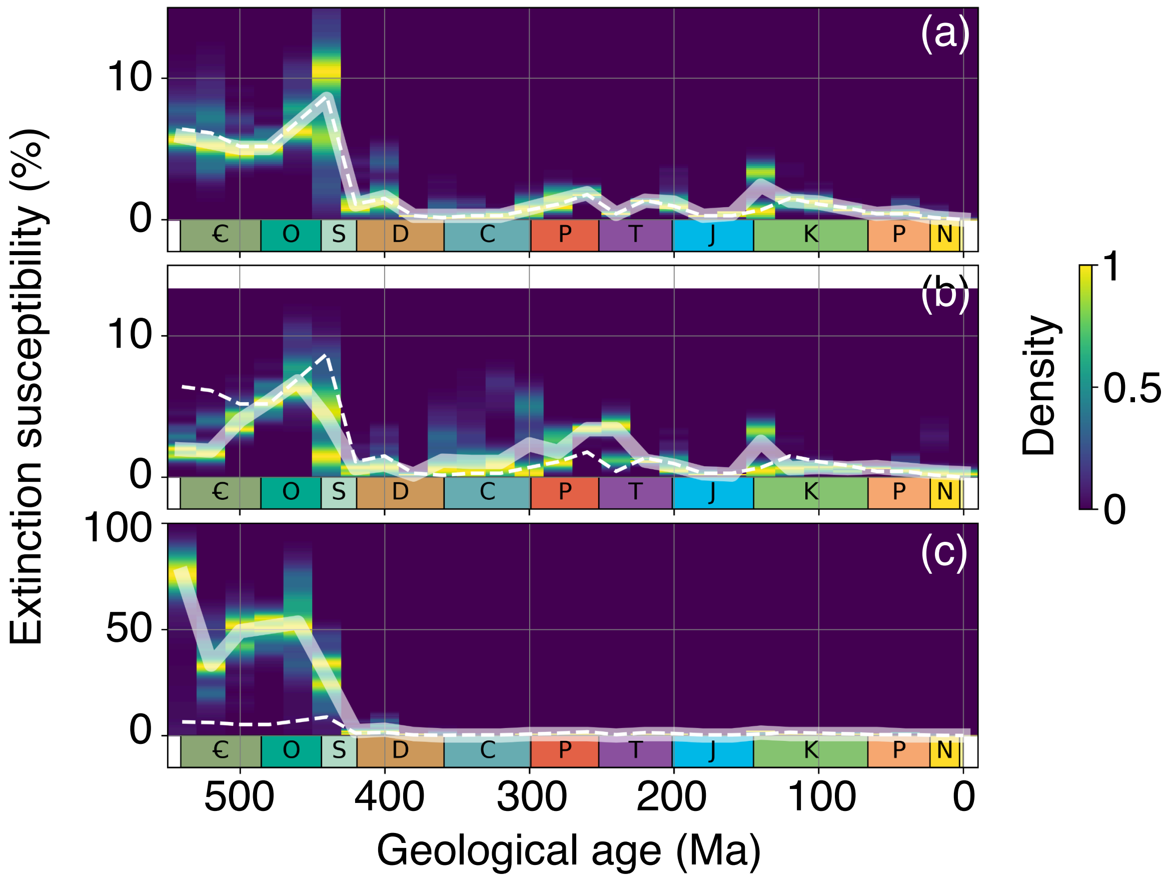

Fig. S3.

Sensitivity test to random sampling protocol. Simulated extinction susceptibility during the Phanerozoic. (a) 'Baseline' simulations with sampling rate fixed at 0.33 (density distribution and thick solid line) and with sampling rate linearly increasing from 0.2 at 540 Ma to 0.8 at 0 Ma (thin dashed line), using 1000 sampling repetitions and sampling pre-warming and post-warming states at different shelf grid points. (b) 'constant SST' simulations with sampling rate fixed at 0.33 (density distribution and solid line, 1000 repetitions using different sampling points). Results of the baseline simulations with sampling rate fixed at 0.33 (1000 repetitions using different sampling points) overlaid for comparison (dashed line). (c) Same as (b) for 'pO_2' simulations. Y-scale differs in the 3 panels. Same as Fig. 3A-C but sampling different (as opposed to identical) shelf grid points in the pre-warming and post-warming states. €: Cambrian, O: Ordovician, S: Silurian, D: Devonian, C: Carboniferous, P: Permian, T: Triassic, J: Jurassic, K: Cretaceous, P: Paleogene, N: Neogene.

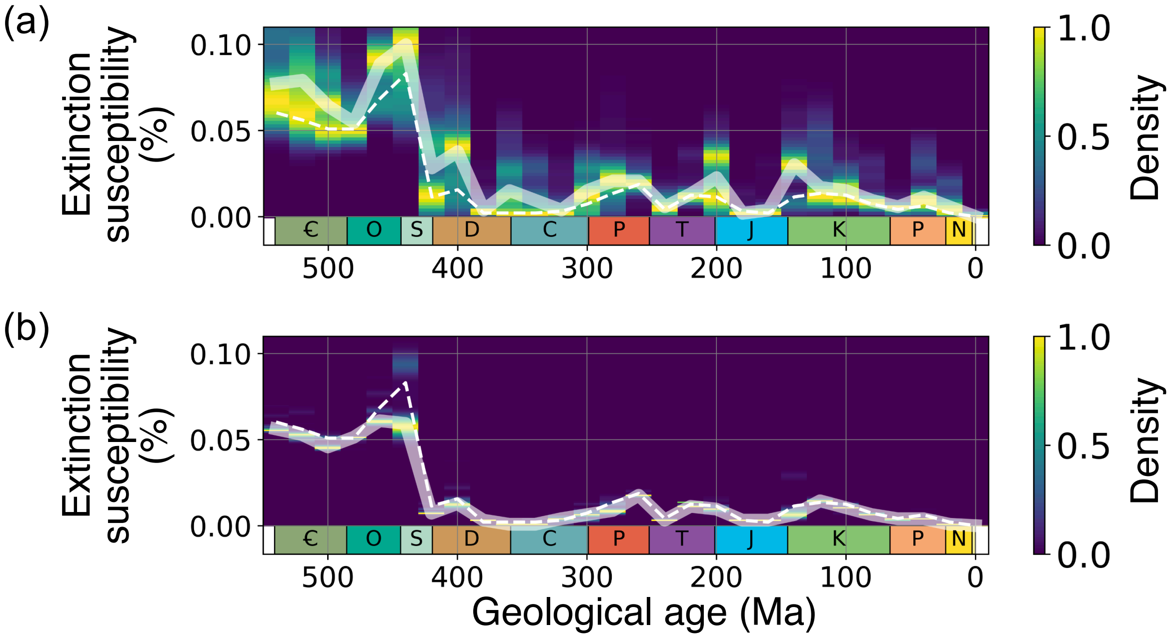

Fig. S4.

Sensitivity test to random sampling rate. Simulated extinction susceptibility during the Phanerozoic. (a) 'Baseline' simulations with sampling rate fixed at 0.1 (density distribution and thick solid line) and 0.33 (thin dashed line, like Fig. 3A). (b) 'Baseline' simulations with sampling rate fixed at 0.75 (density distribution and thick solid line) and 0.33 (thin dashed line, like Fig. 3A). All simulations use 1000 sampling repetitions and sample pre-warming and post-warming states at same shelf grid points. €: Cambrian, O: Ordovician, S: Silurian, D: Devonian, C: Carboniferous, P: Permian, T: Triassic, J: Jurassic, K: Cretaceous, P: Paleogene, N: Neogene.

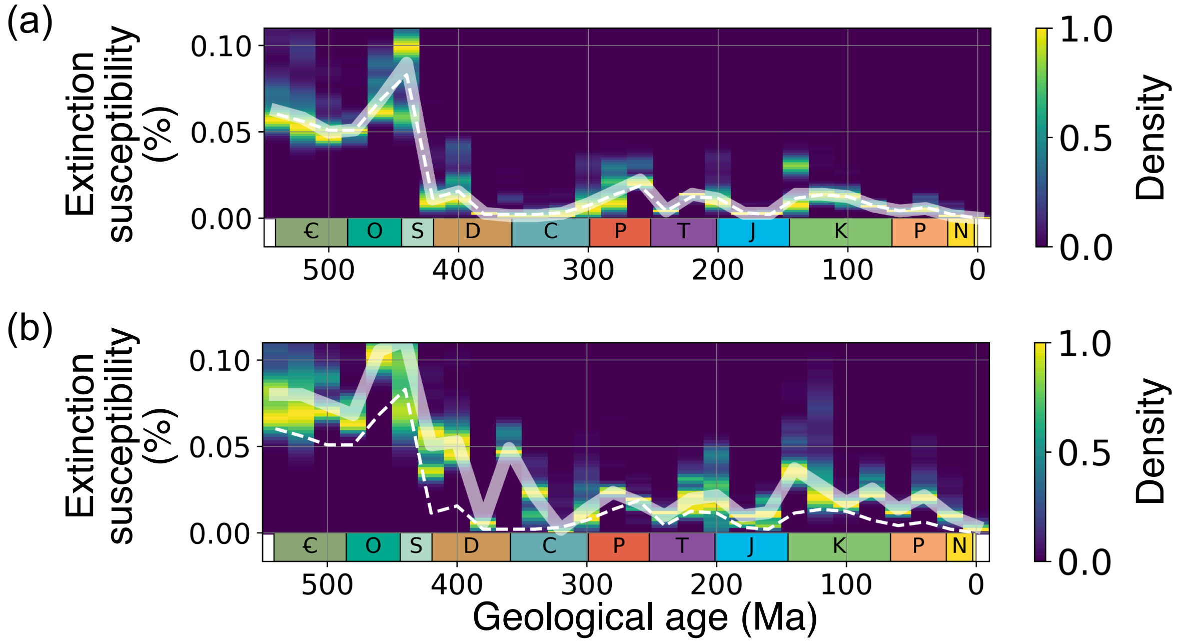

Fig. S5.

Sensitivity test to model depth integration. Simulated extinction susceptibility during the Phanerozoic. (a) 'Baseline' simulations using the surface ocean level only (density distribution and thick solid line) and the 3 upper-ocean levels (thin dashed line, like Fig. 3A). (b) 'Baseline' simulations using the subsurface ocean level only (density distribution and thick solid line) and the 3 upper-ocean levels (thin dashed line, like Fig. 3A). All simulations use a sampling rate of 0.33, 1000 sampling repetitions and sample pre-warming and post-warming states at same shelf grid points. €: Cambrian, O: Ordovician, S: Silurian, D: Devonian, C: Carboniferous, P: Permian, T: Triassic, J: Jurassic, K: Cretaceous, P: Paleogene, N: Neogene.

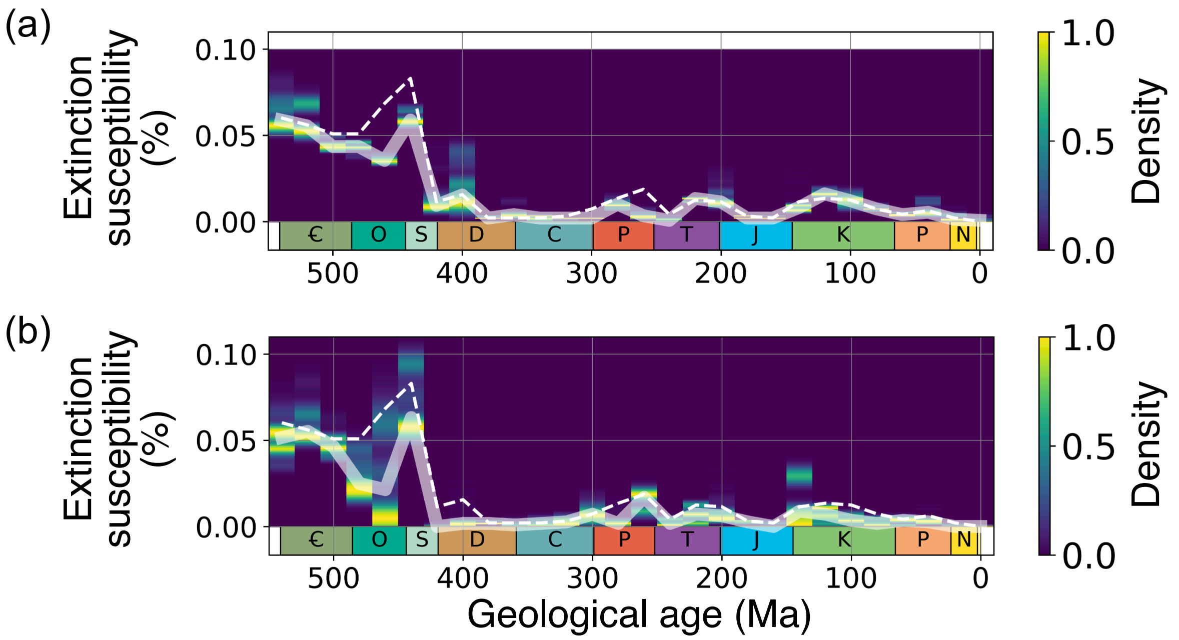

Fig. S6.

Sensitivity test to model spatial domain. Simulated extinction susceptibility during the Phanerozoic. (a) 'Baseline' simulations using the whole upper-ocean domain (density distribution and thick solid line) and shelf points only (thin dashed line, like Fig. 3A). (b) 'Baseline' simulations using shelf points only, using (density distribution and thick solid line) or discarding (thin dashed line, like Fig. 3A) polar grid points. All simulations use a sampling rate of 0.33, 1000 sampling repetitions and sample pre-warming and post-warming states at same grid points. Same as Fig. 3A but using alternative spatial domains. €: Cambrian, O: Ordovician, S: Silurian, D: Devonian, C: Carboniferous, P: Permian, T: Triassic, J: Jurassic, K: Cretaceous, P: Paleogene, N: Neogene.

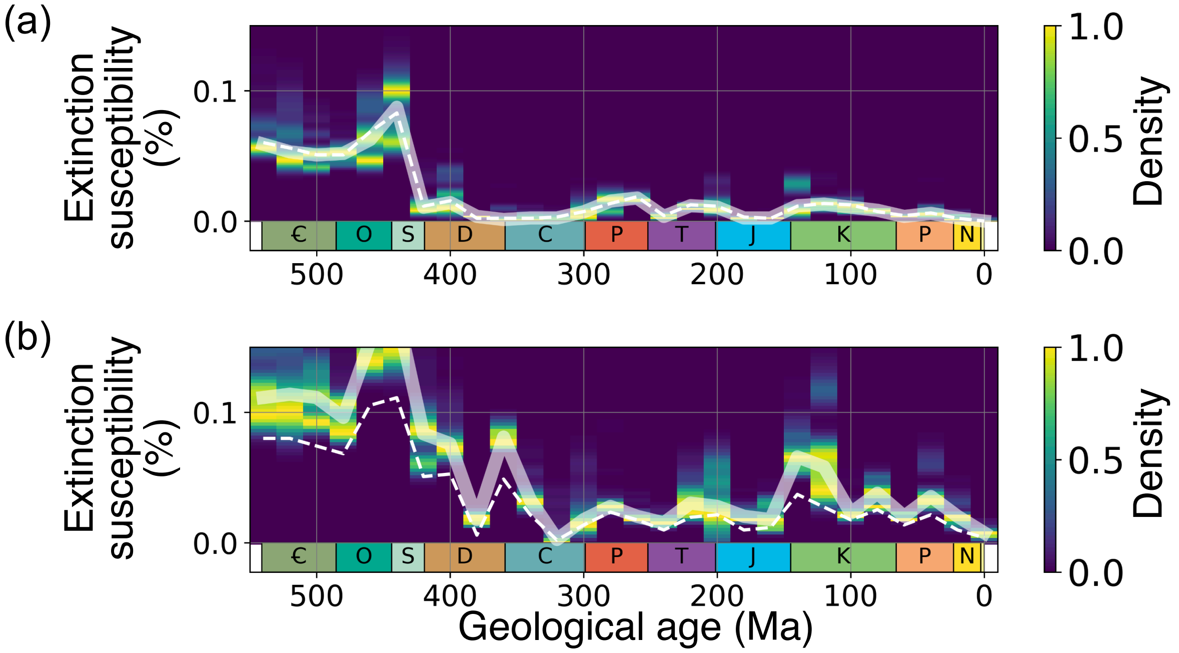

Fig. S7.

Sensitivity test to ocean phosphate inventory. Simulated extinction susceptibility during the Phanerozoic. (a) 'Baseline' simulations considering a 1.5-fold increase in ocean phosphate inventory in response to global warming (density distribution and thick solid line) and a fixed phosphate inventory (thin dashed line, like Fig. 3A). (b) 'Baseline' simulations considering a 1.5-fold increase in ocean phosphate inventory in response to global warming (density distribution and thick solid line) and a fixed phosphate inventory (thin dashed line, like Fig. S5B), but using the subsurface ocean level only (while the upper 3 ocean levels are used in first panel, like in the standard simulations). All simulations use a sampling rate of 0.33, 1000 sampling repetitions and sample pre-warming and post-warming states at same shelf grid points. €: Cambrian, O: Ordovician, S: Silurian, D: Devonian, C: Carboniferous, P: Permian, T: Triassic, J: Jurassic, K: Cretaceous, P: Paleogene, N: Neogene.

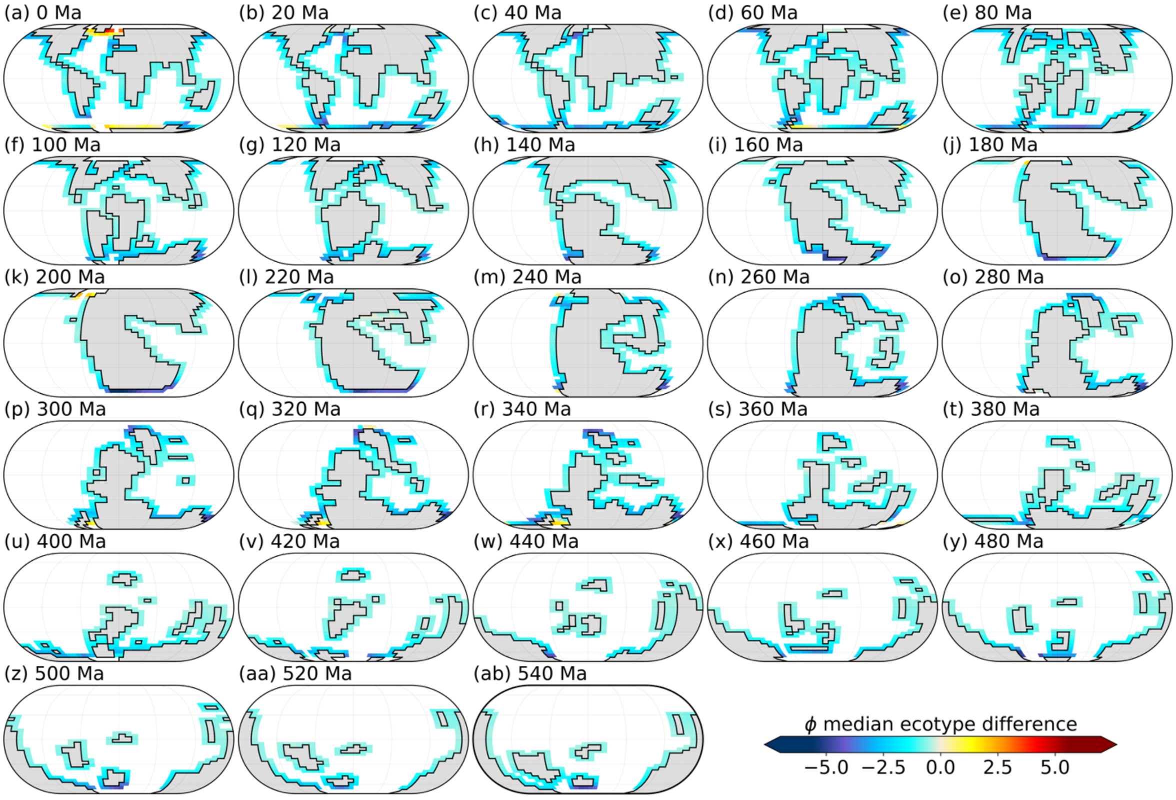

Fig. S8.

Changes in metabolic index values in response to warming. Surface-ocean metabolic index (Φ; see Materials and Methods) change in response to warming in the 'baseline' simulations for an ecophysiotype with median ecophysiological parameters A_0 and E_0. Emerged continental masses are shaded white. Eckert IV projections.

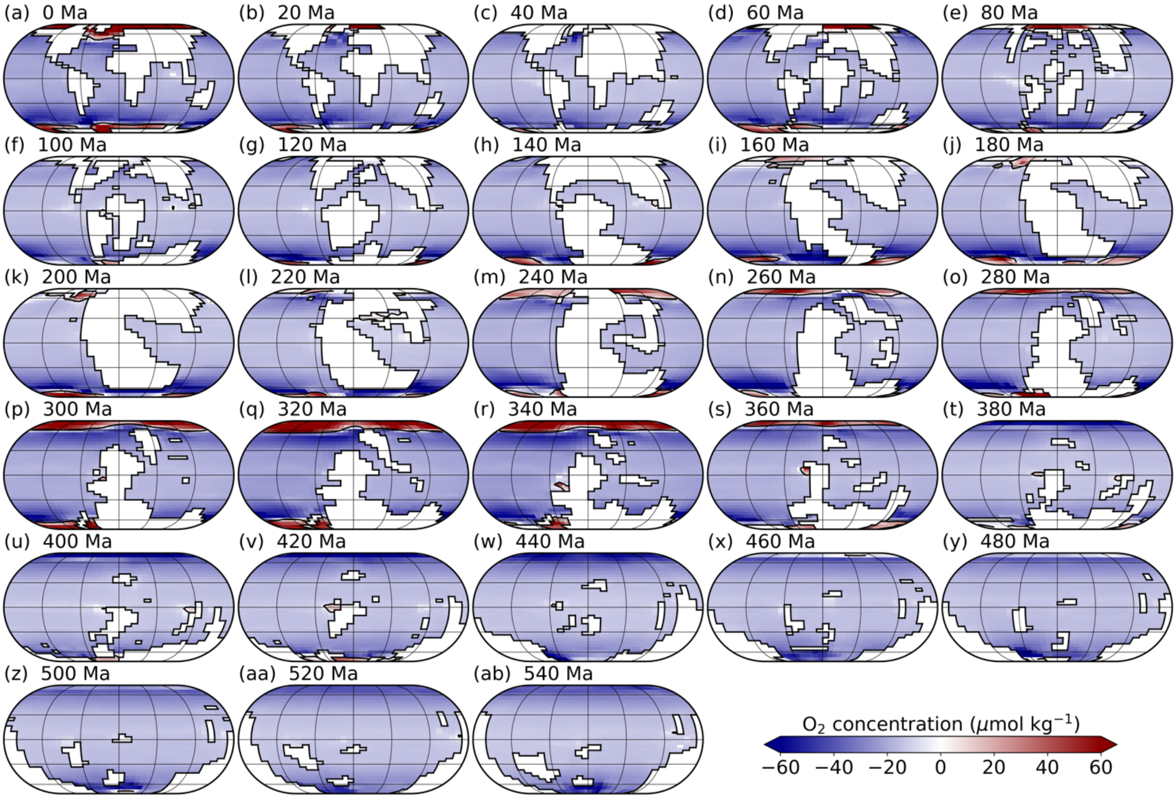

Fig. S9.

Surface-ocean [O_2] change simulated in response to warming in the 'baseline' simulations. Emerged continental masses are shaded white. Eckert IV projections.

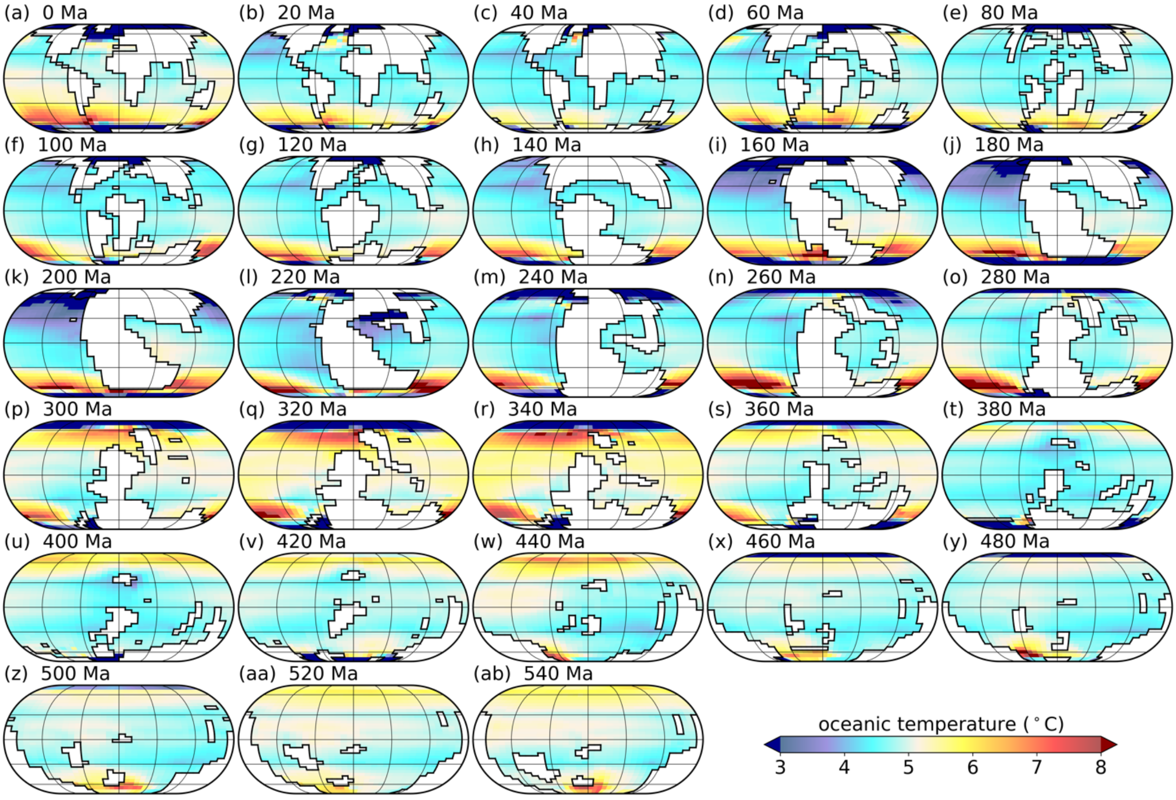

Fig. S10.

Sea-surface temperature change simulated in response to warming in the 'baseline' simulations. Emerged continental masses are shaded white. Eckert IV projections.

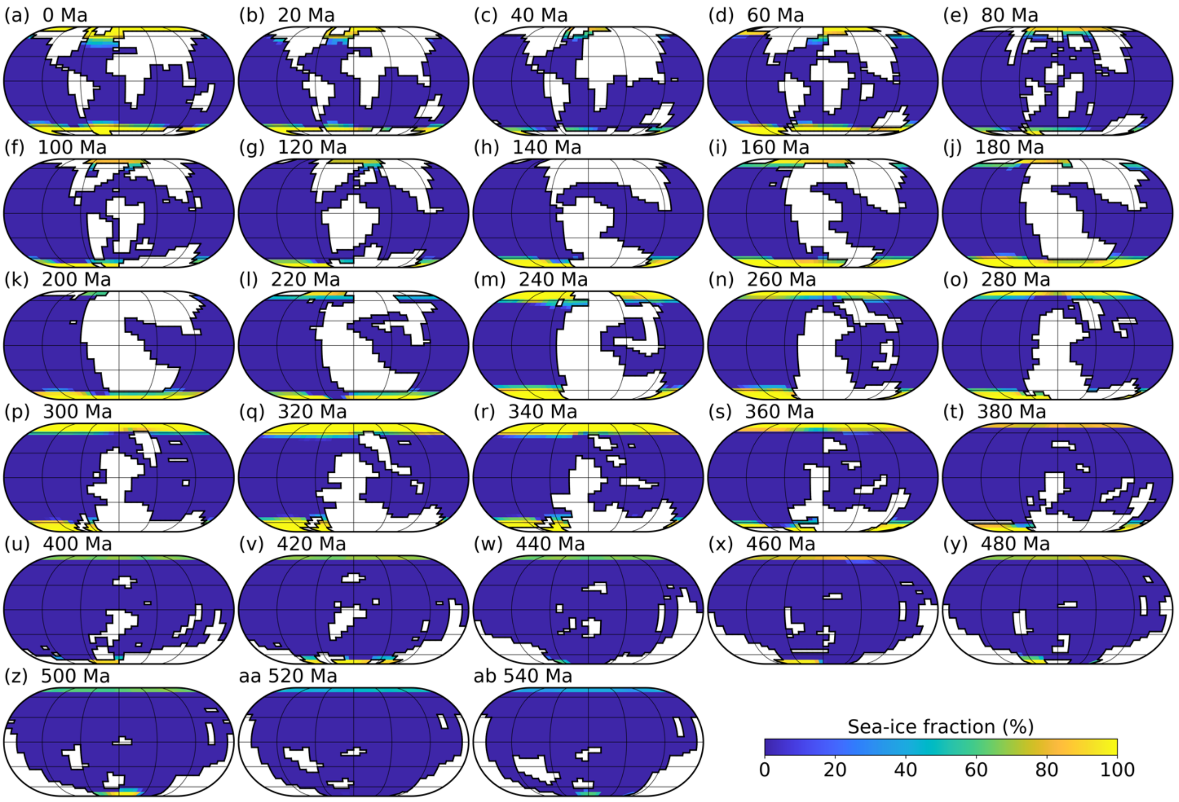

Fig. S11.

Sea-ice fraction in the pre-warming state of the 'baseline' simulations. Emerged continental masses are shaded white. Eckert IV projections.

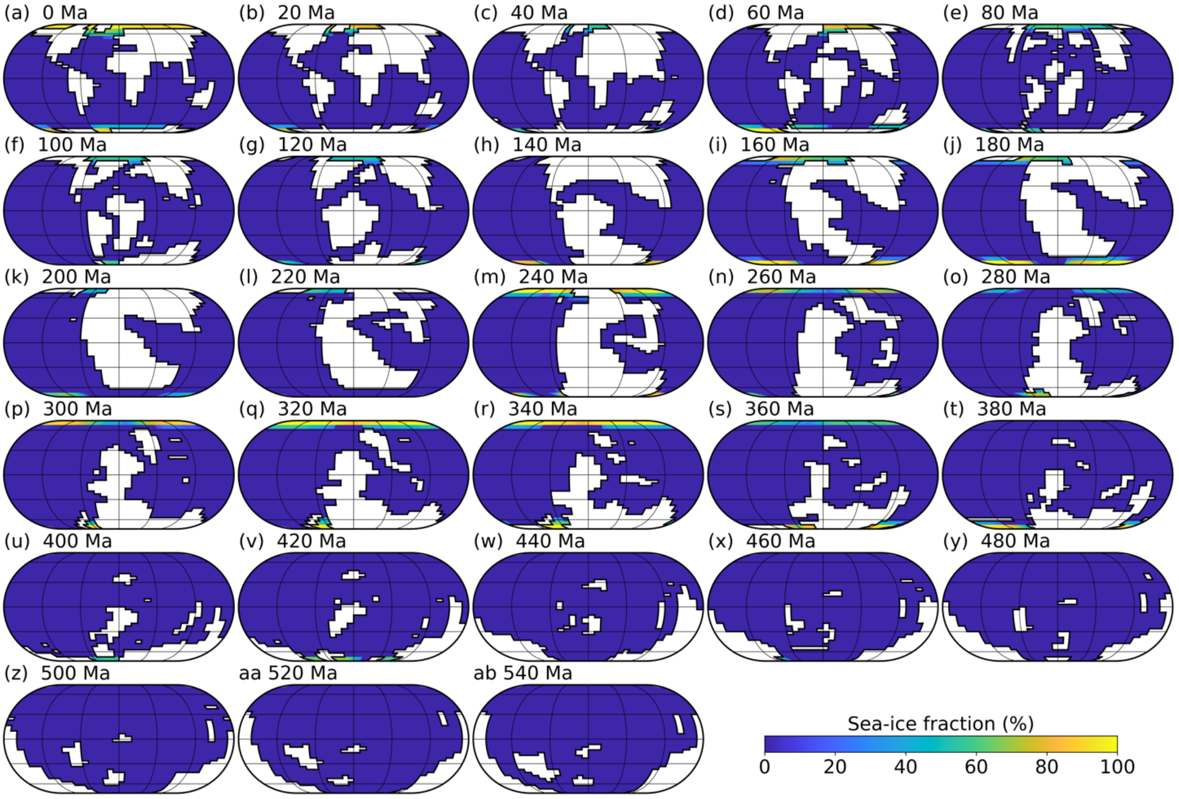

Fig. S12.

Sea-ice fraction in the post-warming state of the 'baseline' simulations. Emerged continental masses are shaded white. Eckert IV projections.

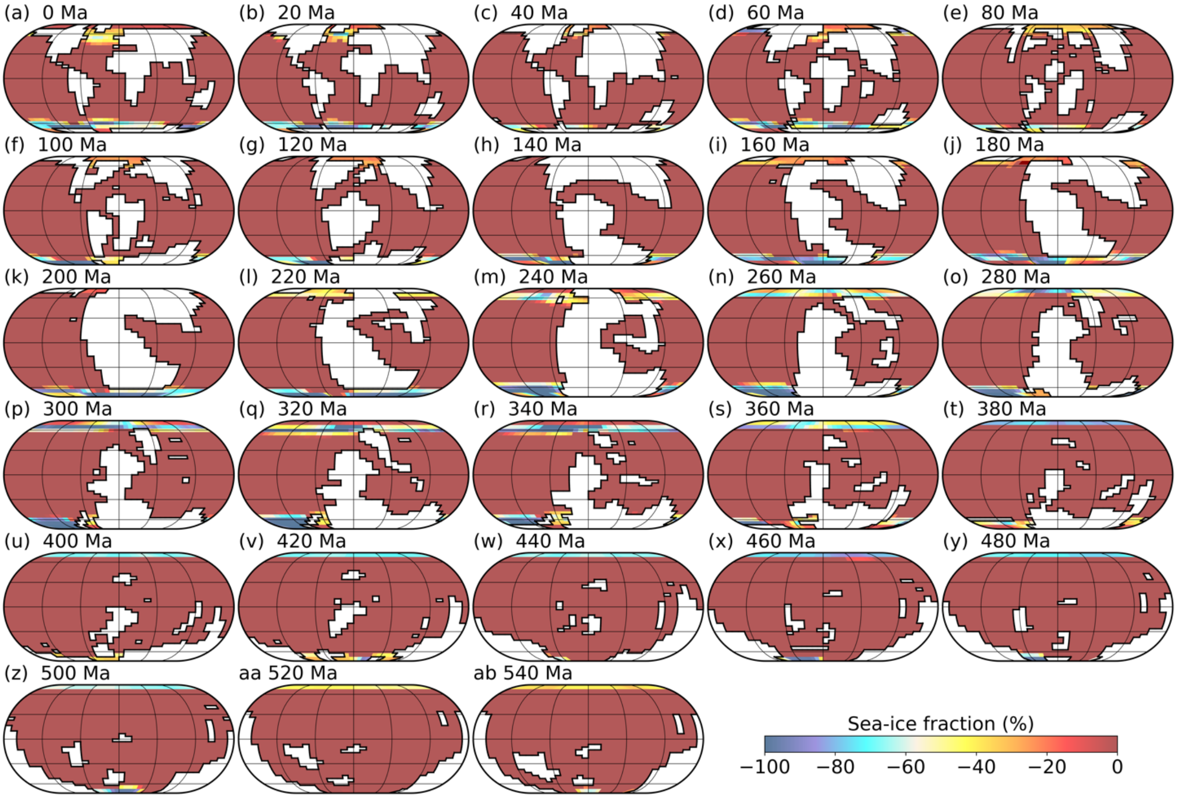

Fig. S13.

Sea-ice fraction change simulated in response to warming in the 'baseline' simulations.
Emerged continental masses are shaded white. Eckert IV projections.

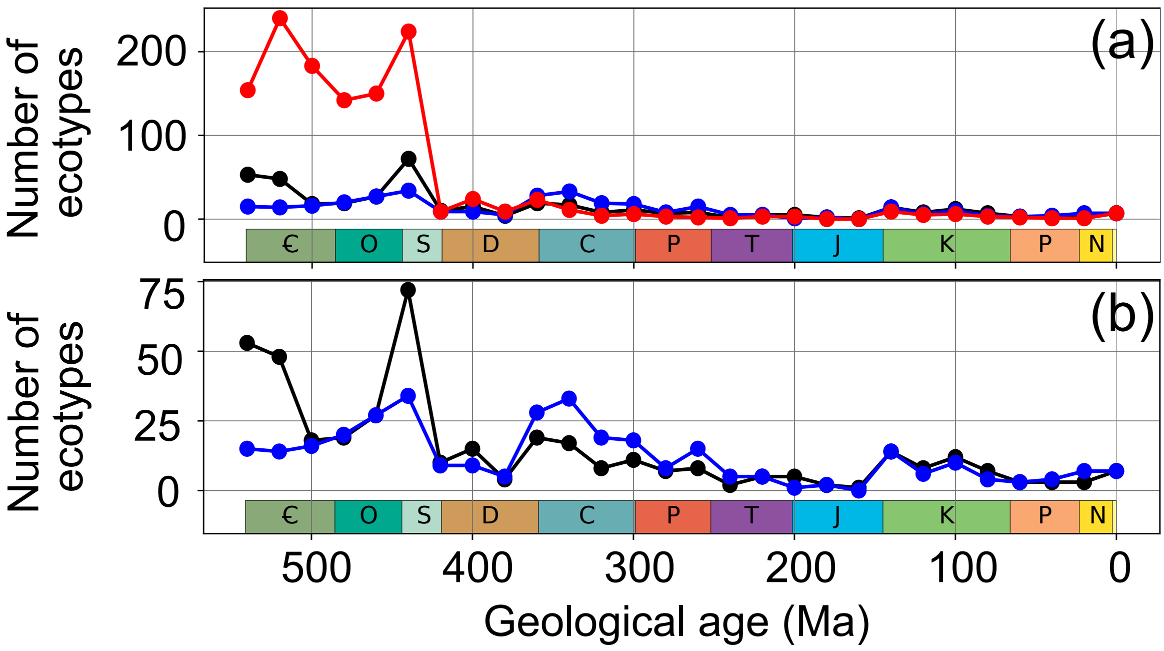

Fig. S14.

Number of ecophysiotypes with limited spatial extent. Number of ecophysiotypes with limited spatial extent (< 10 equal-area model grid cells at any depth level) in the pre-warming state for the 3 series of simulations: 'baseline' (black), 'constant SST' (blue) and 'pO_2' (red). Panel (a) shows results for the 3 simulation series while panel (b) shows results for 'baseline' and 'constant SST' simulations only, for readability (using different Y-scale). €: Cambrian, O: Ordovician, S: Silurian, D: Devonian, C: Carboniferous, P: Permian, T: Triassic, J: Jurassic, K: Cretaceous, P: Paleogene, N: Neogene.

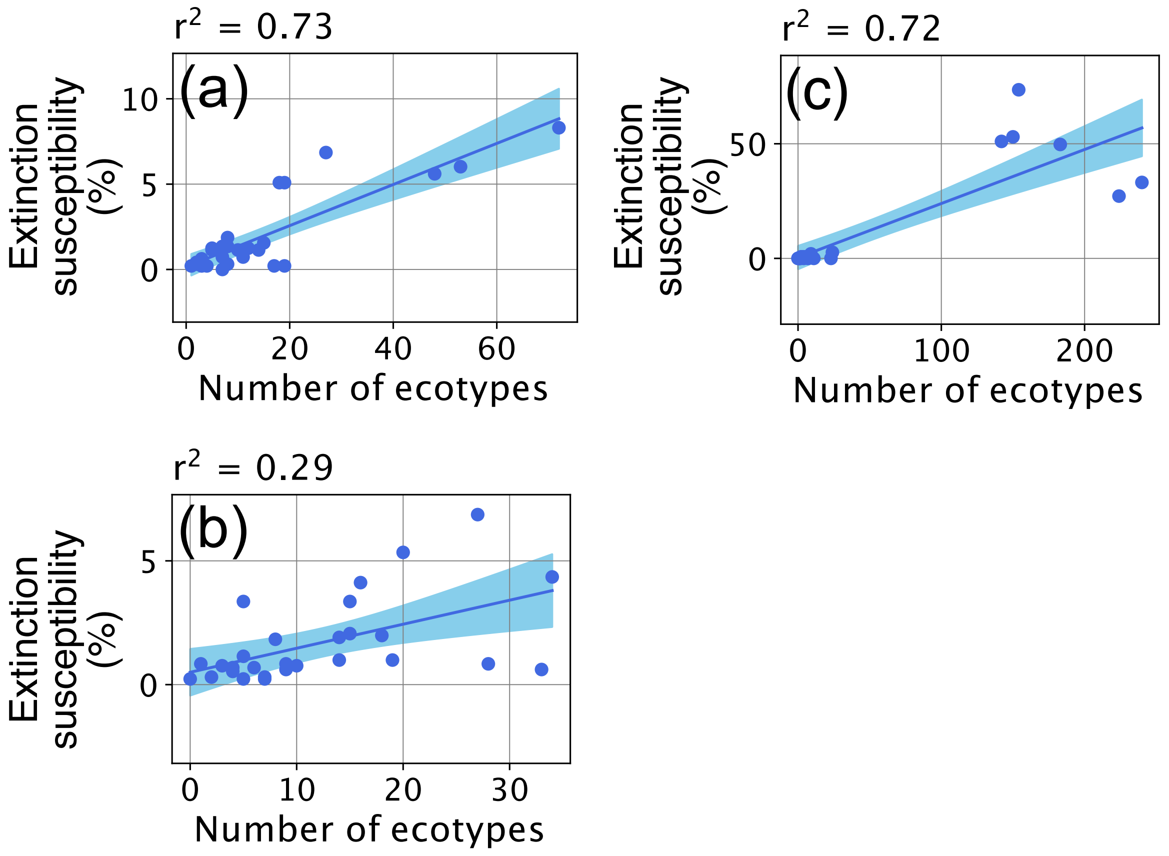

Fig. S15.

Correlation between extinction susceptibility and number of ecotypes with limited spatial extent. Linear correlation between simulated extinction susceptibility (median value calculated by sampling 1000 times at same locations; thick lines in Fig. 3A-C) and number of ecophysiotypes with limited initial spatial extent (< 10 equal-area model grid cells at any depth level in pre-warming state) for (a) 'baseline' simulations, (b) 'constant SST' simulations and (c) 'pO_2' simulations. In each panel, blue points represent each of the 28 time slices and the blue line is the linear correlation line (with 95 % confidence interval shaded blue), the coefficient of which is provided on top of the panel.

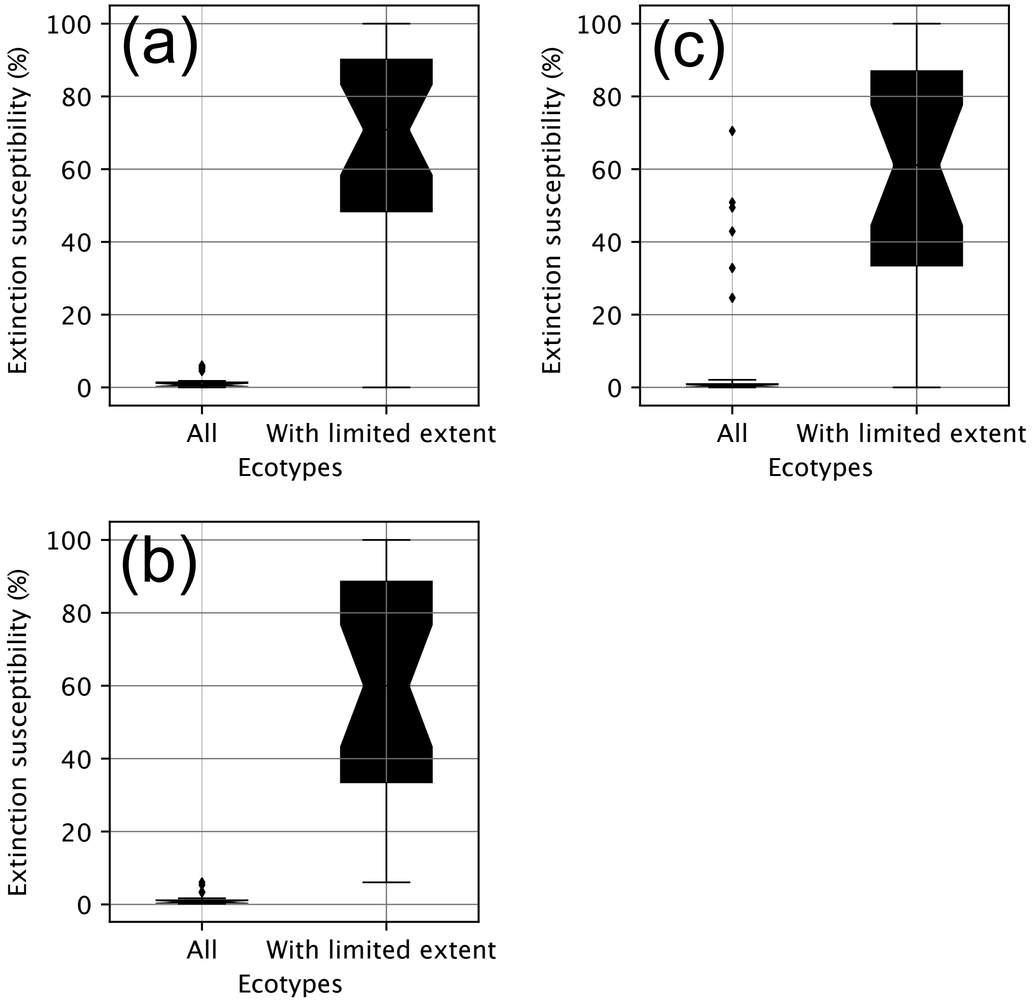

Fig. S16.

Extinction susceptibility for ecotypes with limited spatial extent *vs.* all ecotypes. Extinction susceptibility for ecophysiotypes with limited spatial extent present in the pre-warming state (< 10 equal-area model grid cells at any depth level) *vs.* for all ecophysiotypes. Results are shown for the 3 series of simulations: 'baseline' (a), 'constant SST' (b) and 'pO_2' (c). For each series, the boxplots were calculated based on the individual extinction susceptibilities calculated for each of the 28 time slices studied. Calculated extinction susceptibilities do not account for uncertainties in spatial sampling.

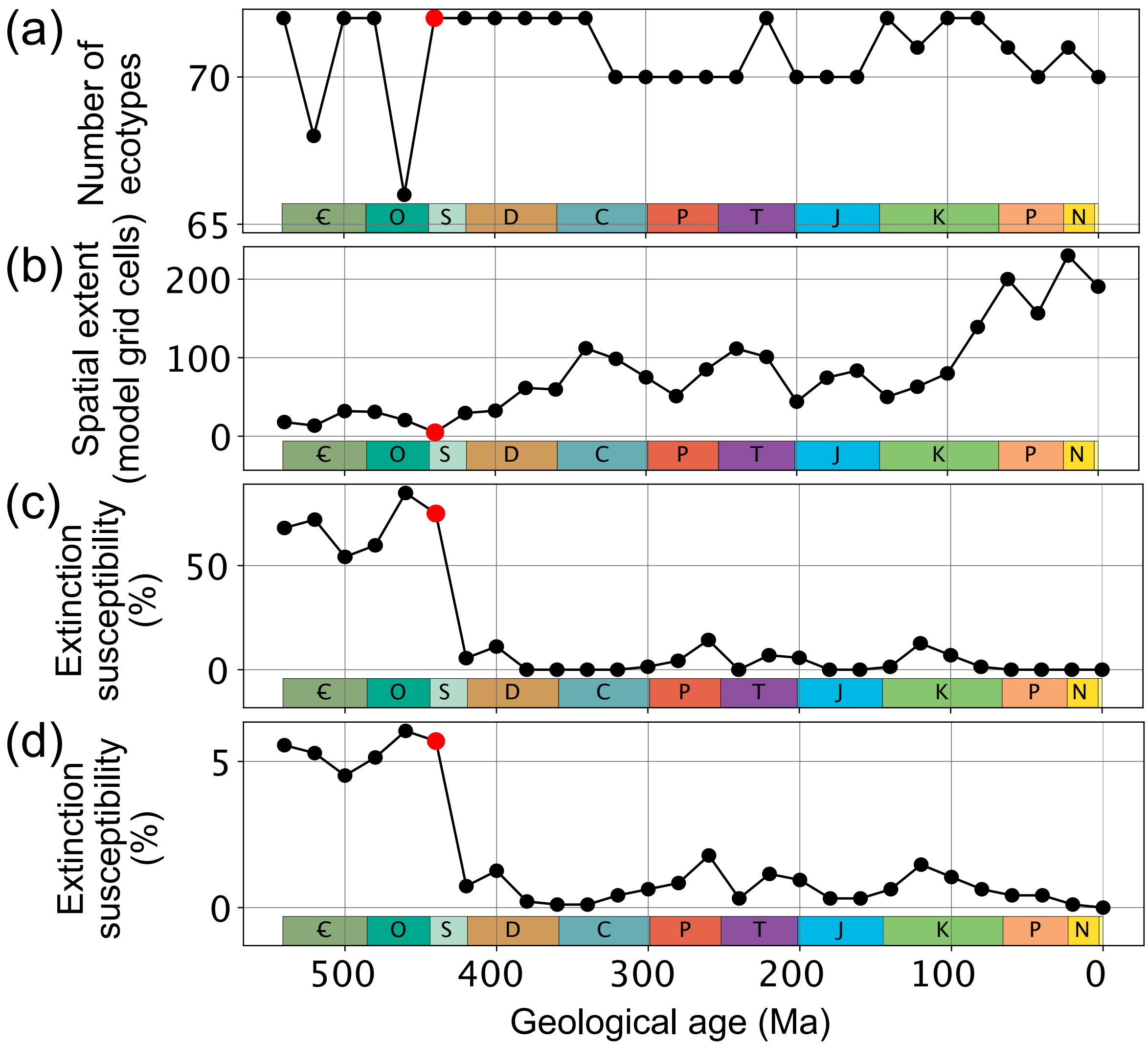

Fig. S17.

Model behavior of ecophysiotypes with limited spatial extent. This figure investigates the behavior of the ecophysiotypes with limited spatial extent (< 10 equal-area model grid cells at any depth level) found in the pre-warming state at 440 Ma (see red point) in the 'baseline' simulations. To that purpose, the ecophysiotypes with limited spatial extent are identified in the pre-warming state for 440 Ma and then, these same ecophysiotypes are studied in all investigated time slices. (a) Number of these ecophysiotypes found in the pre-warming state in the various time slices. (b) Spatial extent of these ecophysiotypes in the pre-warming state in the various time slices, defined as the median number of model equal-area grid cells occupied over the first 3 upper-ocean levels. (c) Extinction susceptibility calculated for these ecophysiotypes in response to warming. (d) Extinction susceptibility for all ecophysiotypes in response to warming. Panels (c) and (d) do not account for uncertainties in sampling. Є: Cambrian, O: Ordovician, S: Silurian, D: Devonian, C: Carboniferous, P: Permian, T: Triassic, J: Jurassic, K: Cretaceous, P: Paleogene, N: Neogene.

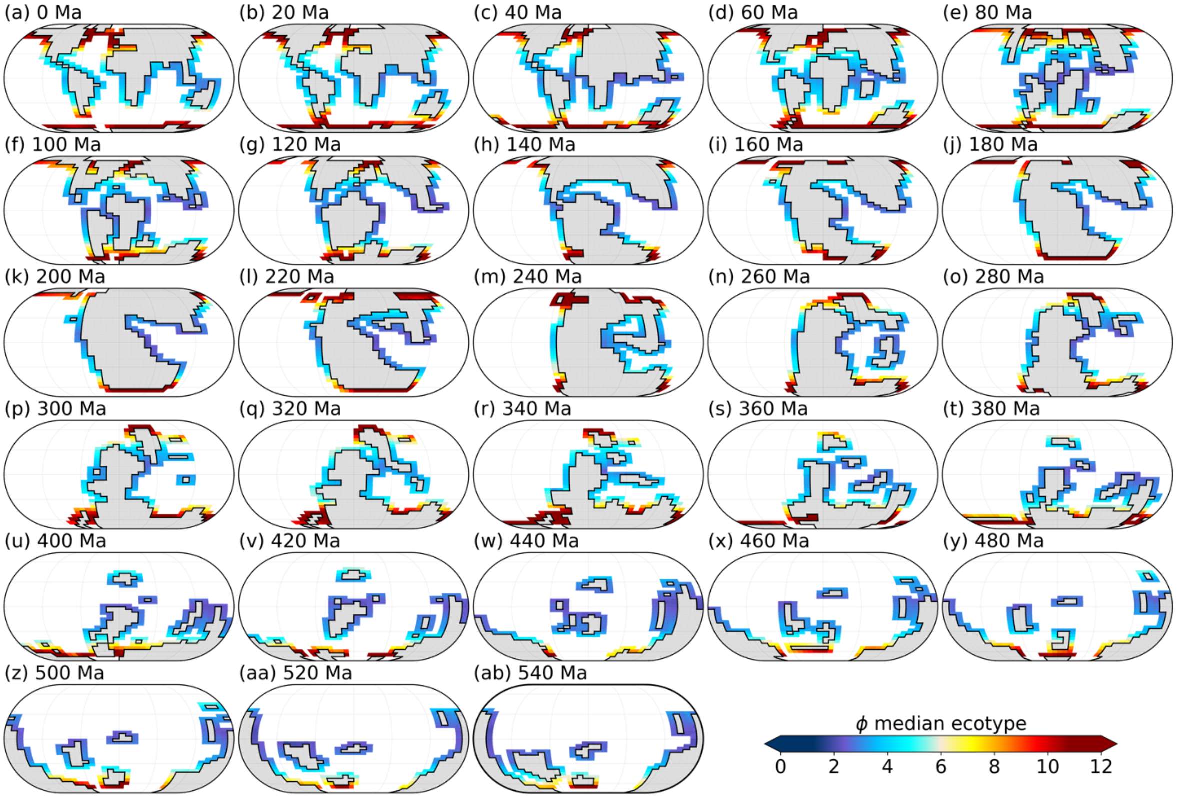

Fig. S18.

Metabolic index in the pre-warming state in the 'baseline' simulations. Surface-ocean metabolic index (Φ; see Materials and Methods) in the pre-warming state in the 'baseline' simulations for an ecophysiotype with median ecophysiological parameters A_0 and E_0. Emerged continental masses are shaded white. The early Paleozoic (540 – 440 Ma) marine environment is spatially heterogeneous at the high latitudes, with strong variations in metabolic index values over small geographical distances (high values being found in only a few grid points, surrounded by much smaller values). Regions with high metabolic index values become spatially more extensive in the Southern Hemisphere from 420 Ma onwards and can be found also in the Northern Hemisphere from 340 Ma onwards. Eckert IV projections.

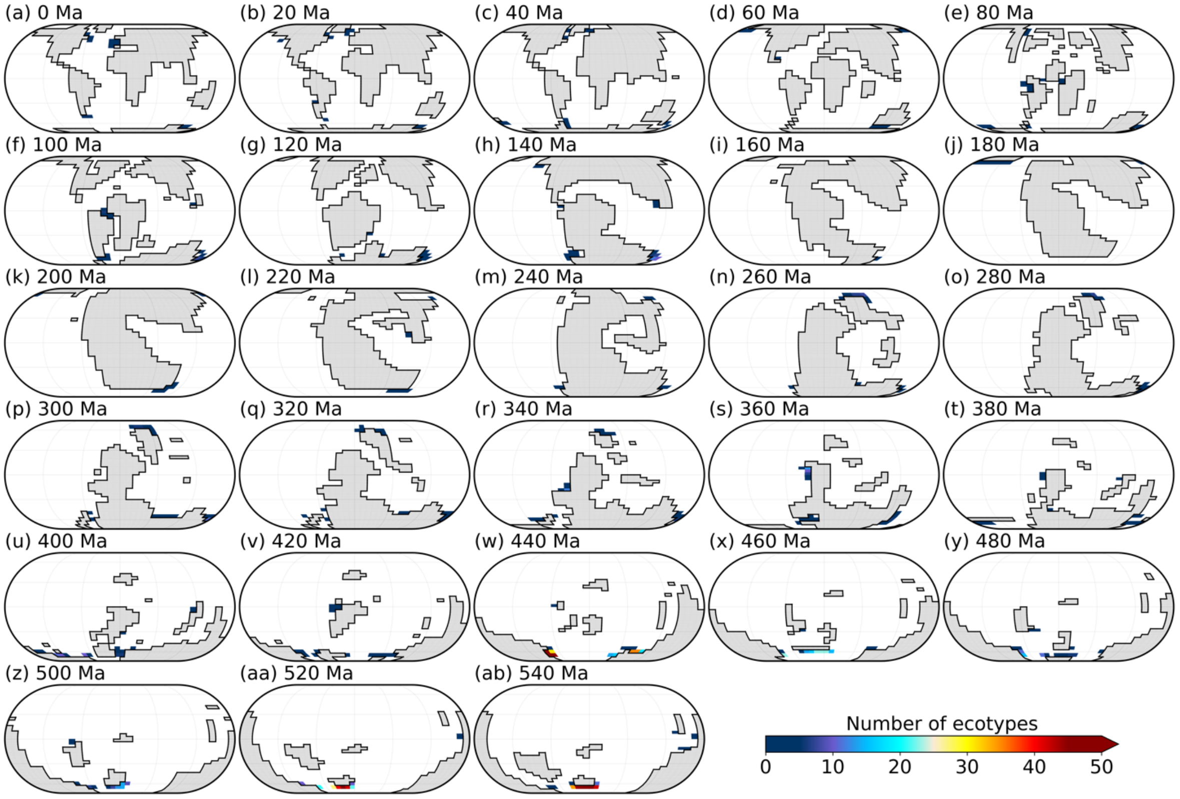

Fig. S19.

Maps of surface-ocean number of ecophysiotypes with limited spatial extent in the 'baseline' simulations. Ecophysiotypes with limited spatial extent are identified as all ecophysiotypes that occupy strictly less than 10 equal-area grid cells in any of the 3 upper ocean levels, in the pre-warming state. Diversity is here calculated at the number of these ecophysiotypes that live in each of the model grid cells. Emerged continental masses are shaded grey. Eckert IV projections.

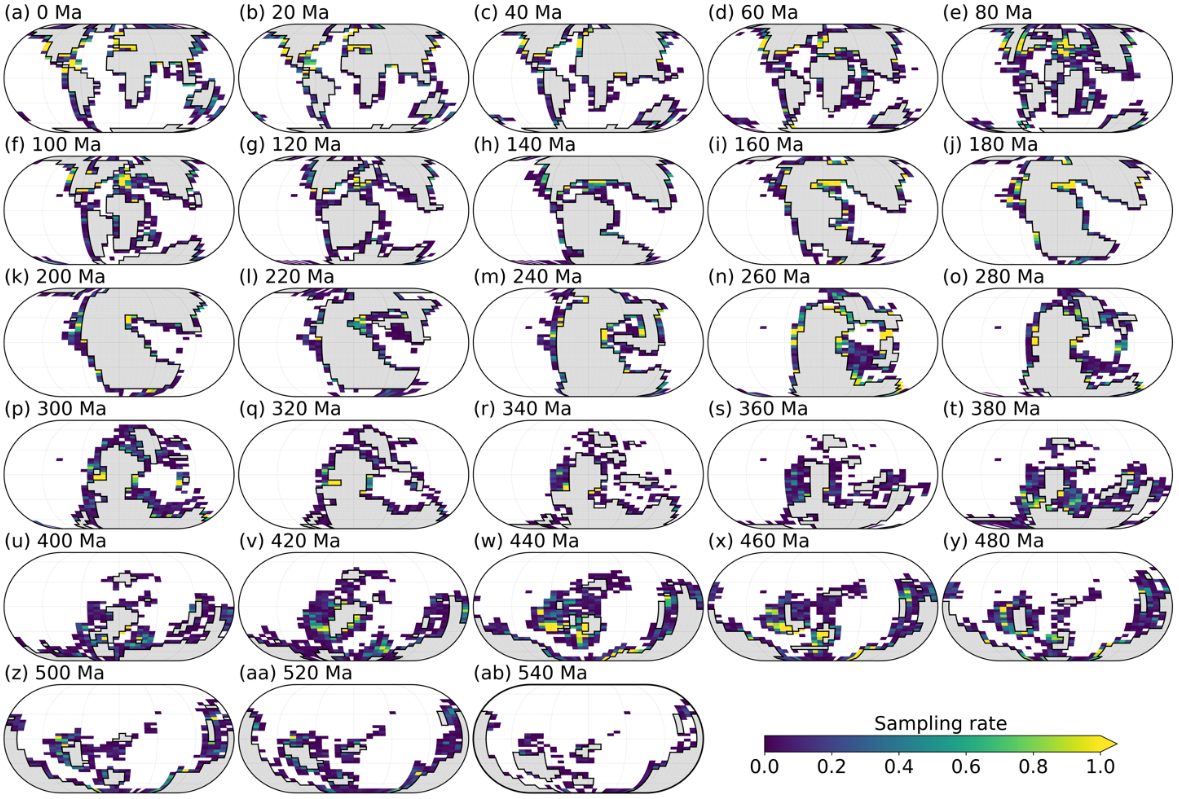

Fig. S20.

Maps of sampling rate derived from the number of collections in our cured PBDB data (Materials and Methods). Points with a sampling rate of 0 (i.e., containing no PBDB collection) are masked. A sampling rate of 1 means that we calculate extinction risk at that location based on the total information contained in the model grid point (i.e., absence or presence of all 1000 ecophysiotypes). Emerged continental masses are shaded grey. Eckert IV projections.

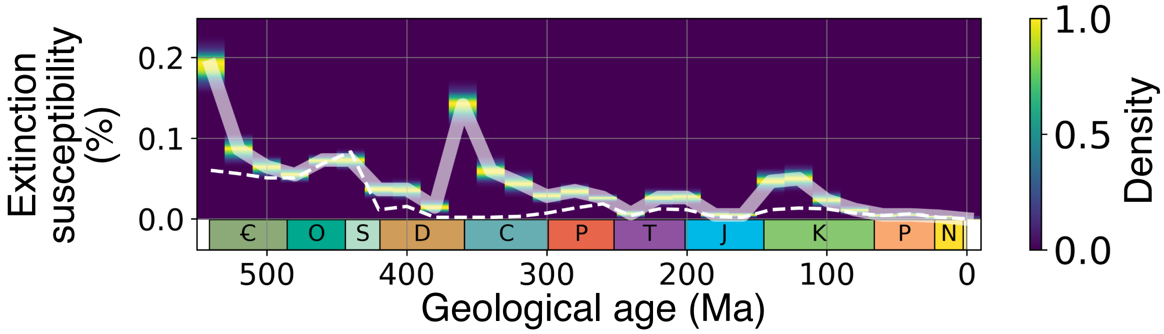

Fig. S21. Extinction susceptibility calculated based on PBDB entries. Same as Fig. 3D but using PBDB entries (instead of collections) for subsampling. €: Cambrian, O: Ordovician, S: Silurian, D: Devonian, C: Carboniferous, P: Permian, T: Triassic, J: Jurassic, K: Cretaceous, P: Paleogene, N: Neogene.
